# The Role of G Protein-Coupled Receptors (GPCRs) and Calcium Signaling in Schizophrenia. Focus on GPCRs Activated by Neurotransmitters and Chemokines

**DOI:** 10.3390/cells10051228

**Published:** 2021-05-17

**Authors:** Tomasz Boczek, Joanna Mackiewicz, Marta Sobolczyk, Julia Wawrzyniak, Malwina Lisek, Bozena Ferenc, Feng Guo, Ludmila Zylinska

**Affiliations:** 1 Department of Molecular Neurochemistry, Faculty of Health Sciences, Medical University of Lodz, 92215 Lodz, Poland; tomasz.boczek@umed.lodz.pl (T.B.); joanna.mackiewicz1@stud.umed.lodz.pl (J.M.); marta.sobolczyk@stud.umed.lodz.pl (M.S.); julia.wawrzyniak@stud.umed.lodz.pl (J.W.); malwina.lisek@umed.lodz.pl (M.L.); bozena.ferenc@umed.lodz.pl (B.F.); 2 Department of Pharmaceutical Toxicology, School of Pharmacy, China Medical University, Shenyang 110122, China; blueforest611@hotmail.com

**Keywords:** schizophrenia, G protein-coupled receptors, neurotransmitters, chemokines, calcium, drug development

## Abstract

Schizophrenia is a common debilitating disease characterized by continuous or relapsing episodes of psychosis. Although the molecular mechanisms underlying this psychiatric illness remain incompletely understood, a growing body of clinical, pharmacological, and genetic evidence suggests that G protein-coupled receptors (GPCRs) play a critical role in disease development, progression, and treatment. This pivotal role is further highlighted by the fact that GPCRs are the most common targets for antipsychotic drugs. The GPCRs activation evokes slow synaptic transmission through several downstream pathways, many of them engaging intracellular Ca^2+^ mobilization. Dysfunctions of the neurotransmitter systems involving the action of GPCRs in the frontal and limbic-related regions are likely to underly the complex picture that includes the whole spectrum of positive and negative schizophrenia symptoms. Therefore, the progress in our understanding of GPCRs function in the control of brain cognitive functions is expected to open new avenues for selective drug development. In this paper, we review and synthesize the recent data regarding the contribution of neurotransmitter-GPCRs signaling to schizophrenia symptomology.

## 1. Introduction

Schizophrenia is one of the most severe psychiatric disorders with the onset typically observed in late-adolescence or early adulthood. While the lifetime prevalence is approximately 1%, regardless of sex, race, or country, the first-degree relatives are ten times more susceptible to develop schizophrenia symptoms than the individuals in the general population [1]. The disease tends to present three main clusters of symptoms: cognitive, positive, and negative. One cluster is usually dominating over another, albeit the prevalence may change over time. The cognitive deficits are often manifested the earliest, long before the onset of the disease in the prodromal stage, and may be visible in the childhood or early adolescence. They can be classified into nonsocial (deficits in verbal fluency, memory, problem solving, speed of processing, visual, and auditory perception) or social, the latter associated with the facial emotion perception and understanding the self and others [2]. The spectrum of positive symptoms includes hallucinations, delusions, suspiciousness, abnormal excitement, and hostility. Among negative symptoms, the most frequently observed are paucity in speech, blunting of affect, loss of motivation, inability to focus on relevant issues, social isolation, apathy, and anhedonia [3]. Negative symptoms are a core component of schizophrenia and are largely responsible for long-term morbidity and poor social functioning of patient with the disorder [4].

The etiology of schizophrenia is unknown. Over 50 years of investigation have demonstrated that the illness does not emerge from a defect in one particular brain region, but rather involves the variety of structural and neurochemical dysfunctions in multiple brain regions. The most extensively studied abnormalities were those in neurotransmitter systems in the brain. For many years, the main theory of schizophrenia was centered on dopamine and its D_2_ receptor. This hypothesis is based on two types of observations: first, antipsychotic medications antagonized dopamine receptors and second, certain drugs such as amphetamine caused psychosis or exacerbated schizophrenic symptoms by enhancing dopamine activity in subcortical and limbic brain regions [5]. This was further supported by brain imaging studies showing increased density of dopamine D_2_ receptors in antipsychotic-free patients [6]. Similarly, both serotonin (5-HT) and norepinephrine have been implicated in disease’s pathophysiology due to a potency of second-generation antipsychotics to antagonize 5-HT and α-adrenergic receptors. The role of other neurotransmitters such as glutamate, GABA, and acetylcholine in the neuropathology of schizophrenia have started to gain a particular attention as the genetic studies linked several genes targeting glutamatergic and cholinergic transmission with elevated risk for schizophrenia [7,8]. Recent studies have also linked the disorder with immune dysfunctions and inflammatory process [9], which may be associated with accelerated aging and greater comorbidity and mortality. The evidence has emerged to suggest that chemokines action, beyond their classical chemotactic functions, may confer majority of the inflammatory aspects of neuro-immune axis. This includes, but is not limited to, previously neglected to direct neurotransmitter like-effects, control of blood–brain barrier permeability, regulation of neurogenesis, neuroendocrine axes, neuronal sprouting, and axonal outgrowth [10,11,12].

A large number of neurotransmitters involved in schizophrenia act through metabotropic G protein-coupled receptors (GPCRs). These receptors mediate slow synaptic transmission by modulating intracellular signal transduction and induction of gene expression to exert antipsychotic action [13,14]. The GPCRs for serotonin, dopamine, adrenaline, and glutamate are traditionally recognized as molecular targets for antipsychotics. However, comprehensive research on GPCR family led to the identification of several allosteric positive or negative modulators or functionally selective compounds targeting different neurotransmitter systems that are now in the center of the concept of biased ligands, which modulate only a given receptor’s downstream signaling [15,16]. The application of this GPCRs-based concept of schizophrenia treatment raises the possibility to implement therapeutically relevant outcomes with neglectable side effects. In this review, we provide a summary of GPCRs-acting neurotransmitters and chemokines and their role in schizophrenia as well as discuss the treatment involving novel mechanisms of GPCR signaling.

## 2. GPCR and Ca^2+^ Signaling

**Figure 1:**
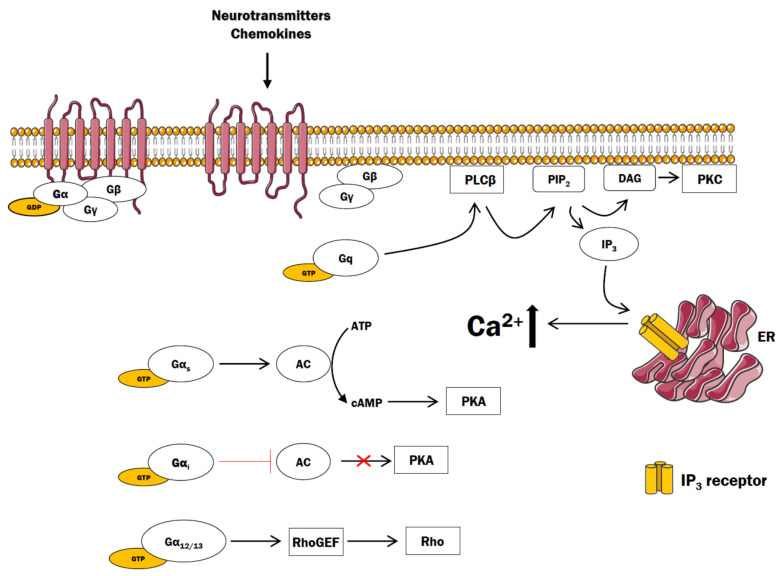
The classical G protein signaling pathways. GDP—guanosine diphosphate, GTP—guanosine triphosphate, AC—adenylyl cyclase, cAMP—cyclic 5′-monophosphate, PKA—protein kinase A, PLC—phospholipase C, PIP_2_—phosphatidylinositol 4,5-bisphosphate, IP_3_—inositol-1,4,5-trisphosphate, PKC—protein kinase C, DAG—diacylglycerol, ER—endoplasmic reticulum.

The G proteins are classified into four main families depending on the α subunit: G_i_, G_s_, G_q_, and G_12/13_. In general terms, G_s_ and G_i_ families affect the intracellular cAMP concentration by regulating adenylyl cyclase activity, whereas G_q_ acts through phospholipase Cβ, and G_12/13_ activates downstream signaling of small GTPases [17] (Figure 1).

Upon ligand binding, the receptor undergoes conformational changes and facilitates the exchange of GDP with GTP in the Gα subunit. Activated Gα-GTP subunit dissociates from heterodimeric Gβγ complex and triggers the activation of key effectors responsible for the generation of second messengers. Depending on the nature of Gα subunit, activation of GPCRs may result in changes in intracellular cAMP, Ca^2+^, diacylglycerol (DAG), or inositol 1,4,5-triphosphate (IP_3_) level that regulate distinct downstream signaling cascades. DAG may bind to and activate protein kinase C (PKC). The Gα_s_ and Gα_i_ exert their effect on protein kinase A (PKA) through the modulation of adenylyl cyclases (ACs) activity, thus regulating the rate of cAMP production. The Gβγ dimer has regulatory and signaling functions, serving as modulator for variety of ion channels and protein kinases, for instance, protein kinase D and phosphatidylinositol-3-kinase [15,18,19]. The IP_3_ diffuses from plasma membrane compartment to the ER where it binds IP_3_ receptors ultimately leading to the release of Ca^2+^ to the cytosol. Emptying ER from calcium ions is detected by stromal interaction molecules (STIM) that are moved to the cell membrane and activate Ca^2+^ release-activated Ca^2+^ (CRAC) channels (formed from ORAI proteins) and transient receptor potential canonical (TRPC) channels [20]. Increased level of Ca^2+^ in cytosol can induce the formation of the Ca^2+^/calmodulin complex, which can exert direct and indirect actions on cell functioning. Up to now, a huge number of proteins in all eukaryotic cells can be regulated by this complex [21]. For example, a direct, short time effect includes activation of phosphatase calcineurin (CaN) that dephosphorylates the family of transcription factor nuclear factor of activated T cell (NFAT) enabling its translocation to the nucleus [22]. Accumulating evidence showed that NFAT is widely expressed in the CNS and plays critical roles in neurological diseases [23,24,25]. Calcineurin is also linked to receptors for several brain transmitters including glutamate, dopamine, and GABA, and plays a key role in the interaction between pro-inflammatory and anti-inflammatory signals [26].

Overactivation of PLC-mediated pathway by elevated cytosolic Ca^2+^ level may induce the oxidative stress by increasing pro-oxidant and decreasing antioxidant potency within cells and tissues. High level of oxidative stress has been reported in peripheral blood cells, neutrophils, platelets, cerebrospinal fluid, and post-mortem brain in patients with schizophrenia [27,28]. Oxidative stress is intimately linked to a variety of pathophysiological processes, such as inflammation, oligodendrocyte abnormalities, and mitochondrial dysfunction [29,30,31]. An important component in brain pathophysiology is Ca^2+^/CaM-regulated nitric oxide synthase (NOS), which synthesizes nitric oxide (NO) and promotes further generation of a number of reactive oxygen and nitrogen species [32,33,34]. Furthermore, an impaired expression and function of redox-sensitive transcriptional factors (i.e., Nrf2, NF-κB,) can escalate toxic cell damages [35,36]. Nuclear factor erythroid 2-related factor 2 (Nrf2) is a translational activating protein that translocates to the nucleus in response to oxidative stress, resulting in increased expression of numerous cytoprotective genes, including genes coding for mitochondrial and non-mitochondrial antioxidant proteins, and has been shown to play a critical role in the pathogenesis of schizophrenia [37,38].

Nowadays, a large number of studies is directed toward understanding the role of GPCRs for individual neurotransmitter systems in the pathology of schizophrenia. This is because the receptors are the site of action of many drugs widely used in clinical practice but are also considered as novel targets for new generation antipsychotics. Moreover, the neuropharmacological observations with antipsychotic drugs targeting GPCRs underpinned the formulation the major hypotheses of schizophrenia origin that implicated the dopaminergic, adrenergic, cholinergic, serotonergic, glutamatergic, GABAergic systems, and neuroinflammatory processes.

## 3. Dopaminergic Receptors

One of the first hypotheses of schizophrenia origin, put forward in the 1960s, stated that the disease is a result of hyperactivity of dopamine (DA) transmission in the brain, particularly in the striatum [39]. In mammalian brain, five dopamine-binding receptors subtypes have been identified, called D1 through D5 [40]. These receptors are divided into two subfamilies: D1-like receptors (D1 and D5) and D2-like receptors (D2, D3, and D4). This classification is based on different pharmacological properties, neuroanatomical distribution, and the activation of downstream signaling pathways [40]. The D1-like receptors are primarily located on the post-synaptic membrane and their high density has been identified in the striatum, nucleus accumbens, prefrontal cortex hippocampus, thalamus, and hypothalamus [40]. The D2-like receptors are expressed both pre- and postsynaptically and their distribution has been demonstrated in key brain regions affected by schizophrenia: the olfactory tubercule, striatum, nucleus accumbens, hippocampus, amygdala, and hypothalamus [40].

**Figure 2:**
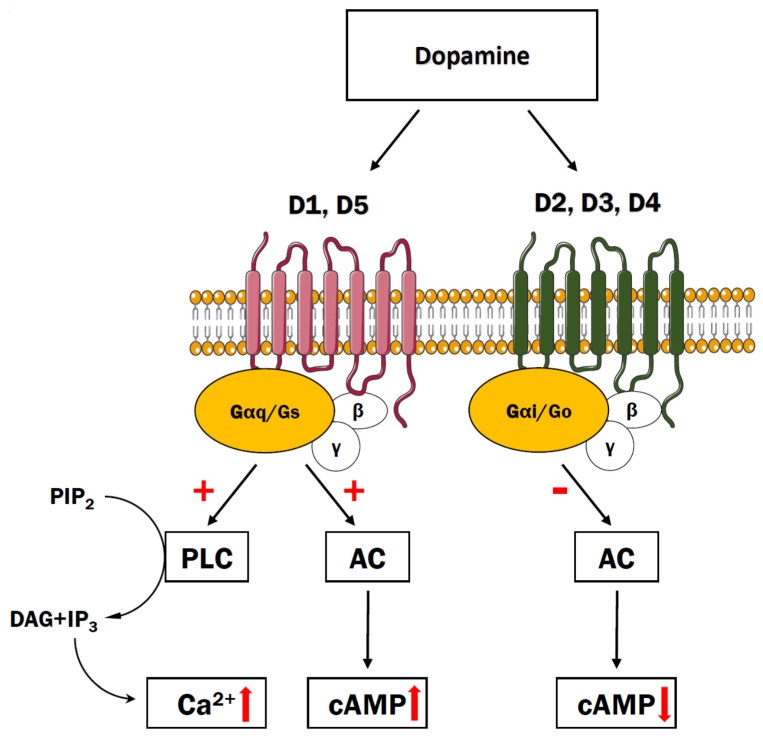
The dopamine system. Binding of dopamine to D1 or D5 receptors activates PLC signaling pathway that triggers Ca^2+^ release from the cisterns of endoplasmic reticulum and has a stimulatory effect on adenylyl cyclase that induces cAMP increase. When acting through D2, D3, or D4 receptors, dopamine exerts an inhibitory effect toward adenylyl cyclase leading to decrease in intracellular cAMP.

Stimulation of the D1-like receptors leads to the activation of the G protein Gα_s_, induction of adenylate cyclase (AC) activity, and subsequent activation of PKA (Figure 2). By contrast, the subfamily of D2-like receptors interact with the Gα_i/o_ of G proteins leading to inhibition of AC activity and reduction of cyclic AMP production [41]. One of the key targets for PKA in the human brain appears to be dopamine- and cAMP-regulated phosphoprotein 32 kDa (DARPP-32), which modulates synaptic transmission, by regulating Na^+^, K^+^, and Ca^2+^ ion channels [42]. DARPP-32 has been found to affect the activity of the receptors for other neurotransmitters such as GABA or acetylcholine [43]. Interestingly, DARPP-32 level was relevantly decreased in the dorsolateral prefrontal cortex (DLPFC) in patients diagnosed with schizophrenia in comparison to controls subjects, which may indicate its involvement in disease-related dysfunction [44].

Abnormal activity of the DA system has been widely implicated in schizophrenia. In schizophrenic patients, the expression of D1 receptors was reduced in prefrontal cortex as determined using PET imaging, which has been linked to development of dysfunction in working memory [45,46]. On the other hand, mRNA levels of D1 receptors were elevated in the temporal and parietal cortex of schizophrenic patients, which may be correlated with auditory hallucinations [47]. Over 40 years of research on the D1 receptor have thoroughly validated its utility as a promising drug target. Recent advancement of new ligands such as drug-like non-catechol D1R agonists and positive allosteric modulators demonstrated that selective modulation of D1 receptors activity may be effective in a treatment of neuropsychiatric disorders including schizophrenia [48].

However, one of the most convincing evidence of disturbances in dopaminergic transmission in schizophrenia comes from the clinical efficacy of first generation and atypical antipsychotics, all being antagonists or partial agonists of D2 receptors [49,50]. The use of these medications is frequently a trade-off between alleviating psychotic symptoms and the risk of sometimes severe adverse effects. The atypical antipsychotics such as clozapine and olanzapine tend to cause metabolic syndrome, whereas first-generation antipsychotics, especially those bound to dopaminergic neuroreceptors, are associated with movement disorders [51]. This indicates the need of searching for novel antidopaminergic agents. Brexpiprazole, for instance, exhibits low risk of D2 receptor sensitization, is well-tolerated, and has low side effects in patients with schizophrenia Moreover, it may have a lower risk for producing rebound symptoms associated with D2 receptor and 5-HT_2A_ receptor sensitization when switching from other antipsychotics such as risperidone [52,53]. The most recently approved, first-in-class antipsychotic—lumateperone—combines the synergy of the drug’s affinity for 5-HT_2A_ receptors at low doses, dose-dependent presynaptic D2 receptors agonism, postsynaptic D2 antagonism, and selectivity to mesolimbic and mesocortical areas for a wide range of symptoms associated with schizophrenia [54].

The involvement of D2 receptors in pathogenesis of schizophrenia is further supported by the data from transgenic mouse models. It has been shown that overexpression of this receptor in the striatum leads to the deficits in inhibitory neurotransmission and dopamine sensitivity in the prefrontal cortex in mouse [55]. Administration of genetic construct encoding enzymes related to the synthesis of dopamine—tyrosine hydroxylase and guanosine triphosphate cyclohydrase—into the substantia nigra pars compacta of adolescent animals resulted in enhancement of dopamine production and appearance of schizophrenia-like behavior [56]. Similarly, administration of dopamine-like drugs such as amphetamine or methylphenidate evoked a hyperlocomotion state in animals and exacerbated psychotic symptoms in schizophrenic patients [57]. It has also been suggested that dopamine D3 receptors may be involved in the regulation of cognitive functions and motor coordination [58]. In line with that, the selective antagonists of these receptors, but not D2 receptors, enhanced social novelty discrimination and novel object recognition in rats, while overall having pro-cognitive effects [59].

Several studies have investigated a possible link between dopaminergic receptor polymorphisms and schizophrenia. A positive correlation was demonstrated between S311C polymorphism of D2 receptor and the response to atypical antipsychotic agents, such as risperidone [60]. The other reports have investigated an association between D3 receptor polymorphism-S9G and occurrence of schizophrenia, however, the results were not consistent [61,62].

The disturbance in dopamine system may be also associated with several mechanisms that involve signaling by other neurotransmitters. For example, Kapur and Seeman demonstrated that pharmacological antagonist of N-methyl-D-aspartate (NMDA) receptor, ketamine, has a strong affinity for D2 receptors [63]. Several studies showed that single dose of ketamine (25 mg/kg, i.p.) increased dopamine release in the prefrontal cortex of rats and repeated administration increased basal dopamine concentration [64,65]. Similarly, MK-801 increased extracellular levels of dopamine and dopamine turnover in the prefrontal cortex and striatum whereas phencyclidine (PCP) in the nucleus accumbens, amygdala, and prefrontal cortex [66]. Although these observations indicate NMDA receptor hypofunction-induced changes in dopaminergic system, they do not explain whether they arise from direct effects over dopamine receptors or indirect action of the drugs via glutamatergic signaling. It has been demonstrated that NMDA dysregulation may provoke psychotic effects at least partially impacting dopamine receptors [67]. There are also indications that dysfunctional dopaminergic signaling in schizophrenia may lie in altered expression or function of dopamine receptor-interacting proteins (DRIPs) [68]. DRIPs play a crucial role in the regulation of intracellular activity of individual dopaminergic receptors in the brain, e.g., their biosynthesis, membrane localization, and signaling [68]. It was reported that one of DRIPs, neuronal calcium sensor I (NCS-1) was upregulated in the DLPFC of schizophrenic brain [69]. The effect of interaction between the D2 receptor and NCS-1 is a control of receptor desensitization and its half-life in the plasma membrane after ligand biding [69]. Such specific relationship between D2 receptor and NCS-1 indicates the crucial role of DRIPs in the regulation of dopamine receptors density and provides a link between abnormalities in the brain dopamine system and defects in Ca^2+^ homeostasis in schizophrenia.

Nonetheless, all the studies done in preclinical models and in humans collectively suggest that the dysregulation of neurotransmitter systems in the pathophysiology of this disorder is significantly more complex and not limited to only abnormalities in the expression and functioning of dopamine receptors.

## 4. Adrenergic Receptors

**Figure 3:**
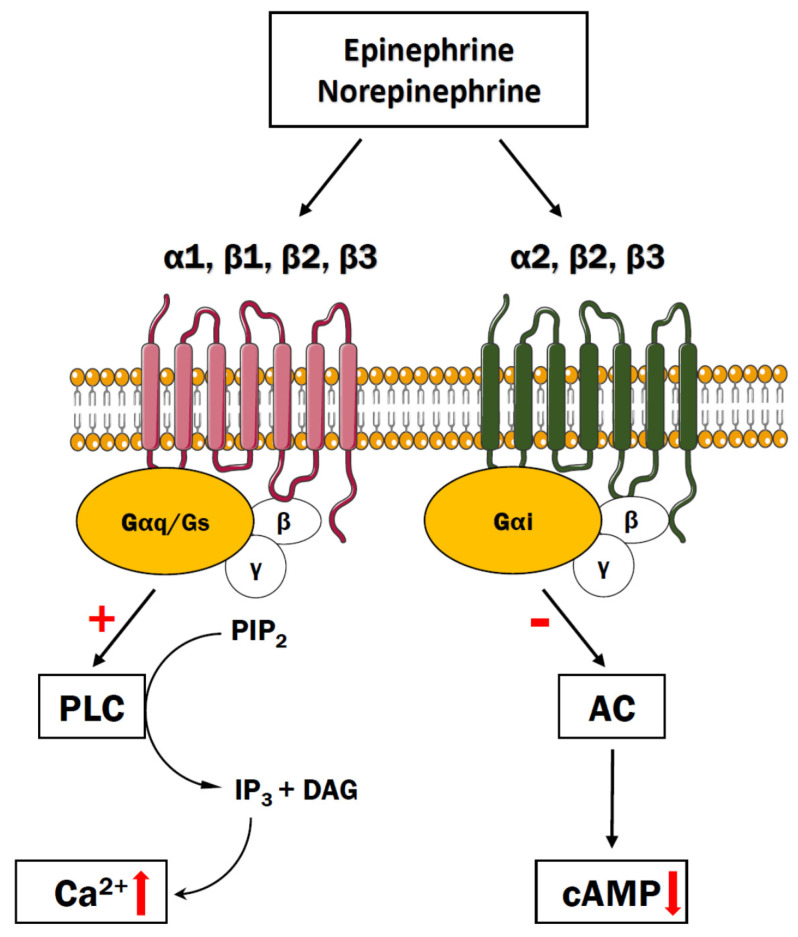
The epinephrine/norepinephrine system. The ligand (epinephrine or norepinephrine) binds adrenergic receptors that are coupled with either activation of PLC signaling resulting in release of Ca^2+^ from the ER or inhibition of adenylyl cyclase, thus decreasing cAMP concentration.

It is commonly known that norepinephrine (NE), also called noradrenaline (NA), as widespread neuromodulator of all cell types in the CNS, orchestrates brain functions, including arousal, stress responses, anxiety, executive control, and also memory consolidation by transmitting its biological signals via α-and β-adrenergic receptors (ARs) [70]. ARs are classified into three groups: α1 (α1A, α1B, α1D), α2 (α2A, α2B, α2C), and β (β1, β2, β3) receptors, all of which are members of the G-protein coupled receptor family but exhibit distinct physiological and pharmacological profiles (Figure 3). The α1 receptors through the Gq signaling pathway increase PLC activity and generate IP3 and DAG to amplify intracellular calcium mobilization [71]. All three β-AR subtypes are prototypic Gs coupled receptors and their stimulation affects intracellular cAMP accumulation and PKA activation [72,73]. In addition, β2 and β3 receptors may couple to Gi protein and influence ERK/MAPK pathway [74], whereas stimulation of Gi/o-coupled α2-ARs suppresses intracellular cAMP signaling and attenuates calcium release, thus inhibiting signal transduction [75]. The ARs are mainly found post-synaptically but α2- and β2 receptors can also exert autoreceptor function at presynaptic terminals of noradrenergic neurons [76,77]. The signal transduction of the NE system in neurons has been extensively reviewed elsewhere [78].

In general terms, the positive symptoms of schizophrenia are exacerbated by selective and indirect noradrenaline receptor agonists such as ephedrine, clonidine, and desipramine, while antagonists, such as yohimbine, propranolol, and oxypertine may ameliorate these symptoms [79]. Although no specific mechanism has yet been confirmed, growing body of evidence indicates that NE signaling through α-AR can contribute to cognitive deficits observed in schizophrenia [80].

It is believed that moderate levels of NE engage high affinity postsynaptic α2-ARs, whereas increased concentrations of this catecholamine, probably released from the locus coeruleus (LC) during stress, impair PFC cognitive function via α1-adrenoceptors [81]. Birnbaum and colleagues observed that administration of potent activator of PKC or indirect stimulation of PKC with α1R agonist can result in a loss of prefrontal cortical regulation involving disrupted cognitive performance and spatial working memory in rats and monkeys [82]. From a pharmacological perspective, specific α2-AR agonists, administered alone or in combination with antipsychotics may enhance neurocognitive functions but also reduce positive and even negative schizophrenia symptoms leading to potentially high clinical relevance for treatment of this disorder. For instance, administration of clonidine to patients with schizophrenia improved stimulus filtering by normalization of both their sensory gating (P50) and sensorimotor gating (PPI) deficits to such levels that were not significantly different from levels of healthy controls [83,84]. Interestingly, the NE system can modulate PPI independently of 5-HT_2A_ neurotransmission and even compensate deficiency of serotonergic system, which seems to be evolutionary advantageous for maintaining enhanced protection against sensimotor gating impairments [85]. Likewise, the manipulation of noradrenergic activity by guanfacine, another α2 receptor agonist, ameliorated cognitive impairments of schizophrenic patients when used as an adjunctive treatment with neuroleptics [86].

Both NE and DA are important components of the arousal systems and their complementary action is needed for proper PFC function [87]. The high levels of D1 stimulation has been demonstrated to increase the production of cAMP, thereby opening hyperpolarization-activated cyclic nucleotide-gated (HCN) cation channels near the synapse and detuning of spatial information processing [88]. In schizophrenia, disturbed stimulation of α2-AR located on the apical dendrites of cortical pyramidal cells may affect dynamics of the HCN channels in cortical pyramidal cells leading to increased hyperpolarization-activated currents and reduced apical amplification [89,90]. As a result, G_s_-mediated excessive cAMP upregulation, which has also been observed in hippocampal CA1 pyramidal cells via noradrenergic suppression, may reduce neuronal firing in the PFC leading to impairing cognitive operations [91]. In an animal study, α2A-adrenoceptor inhibition of cAMP signaling via guanfacine blocked the opening of HCN channels, strengthening the connectivity of the PFC networks related to WM [92,93]. Numerous reports have highlighted the potential involvement of the β-adrenergic receptor in memory consolidation, in particular, toward modulating hippocampal long-term potentiation (LTP) [94], and behavioral memory of mammals through cAMP-PKA signaling [94,95]. However, there is currently insufficient evidence regarding the effectiveness of beta blockers as an adjuvant therapy for the treatment of schizophrenia as reviewed by Cochrane and coworkers [96].

Treatment of patients with adjunctive antidepressants that act on NE activity, for instance duloxetine or mirtazapine, enhanced beneficial effects of atypical antipsychotics (clozapine, risperidone) and relieved negative symptoms of schizophrenia supporting the role of this neurotransmitter in the disease development [97,98,99]. However, recent work has uncovered that haloperidol, risperidone, olanzapine, and clozapine may potently regulate peripheral NE, which may be relevant to drug metabolism-related side effects, e.g., hyperglycemia [100].

Finally, single nucleotide polymorphisms (SNPs) can be also implicated in the etiology of schizophrenia: two SNPs in the promoter region of the α1A-adrenergic receptor (ADRA1A) gene [101], or interactive effect of α2A-adrenergic receptor (ADRA2A) gene polymorphism and methylenetetrahydrofolate reductase (MTHFR) gene polymorphism [102], which may additionally aggravate the low-dopamine state [103].

## 5. Cholinergic Receptors

**Figure 4:**
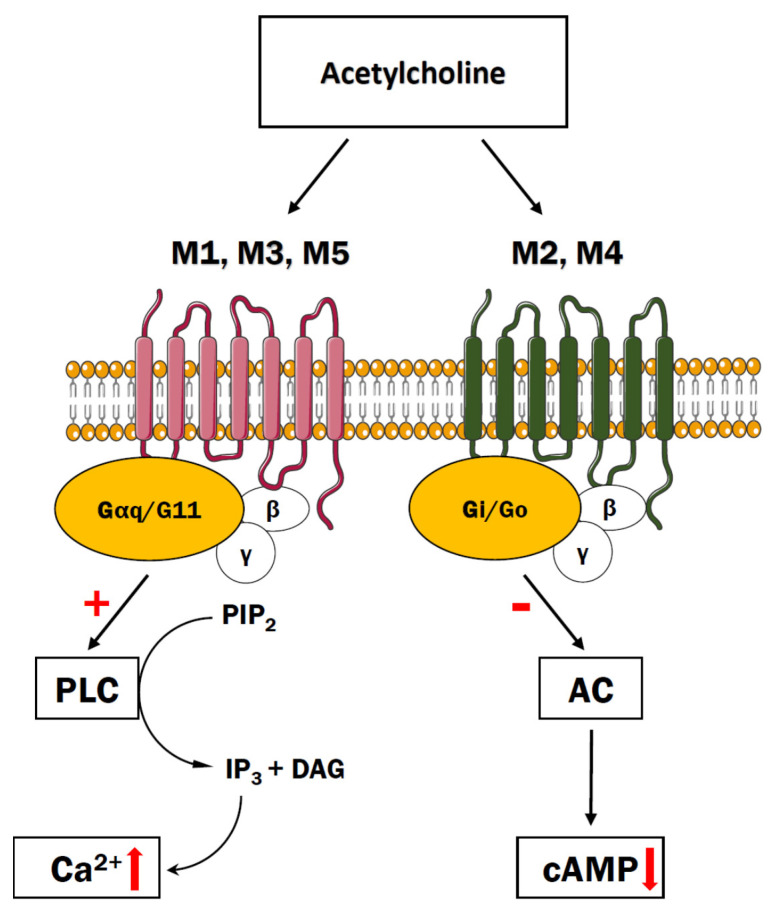
The cholinergic system. The ligand (acetylcholine, carbachol, pilocarpine, etc.) binds muscarinic receptors (M1, M3, M5) to stimulate Ca^2+^ release from the ER via PLC-dependent signaling pathway or inhibits adenylyl cyclase when bound to M2 or M4 receptors.

Muscarinic acetylcholine receptors (mAChRs) are metabotropic receptors that become activated upon binding of neurotransmitter acetylcholine (ACh). Upon activation of the neuron, ACh is released from the synaptic vesicles into the synaptic cleft where it binds to presynaptic and postsynaptic receptors or is inactivated by the enzyme cholinesterase [104]. There are five subtypes of muscarinic receptors, designated as M_1_–M_5_, that can be further subdivided into two groups depending on their functional properties [105]. Stimulation of M_1_, M_3_, and M_5_ receptors, that are expressed postsynaptically across many brain regions and coupled to G_i_/_o_ G-type proteins, initiates the cascade of PLC-dependent reactions related to formation of DAG and IP_3_ (Figure 4). The M_1_ receptor is a predominant subtype detected mainly in cortical and hippocampal neurons whereas neuronal M_3_ and M_5_ subtypes are present at low levels and their role is relatively little known. By contrast, M_2_ and M_4_ muscarinic receptors interact with G_i_ and G_o_-type G proteins and negatively influence adenylyl cyclase, thus inhibiting formation of cAMP [106,107]. In the cerebral cortex and hippocampus, M_2_ receptors have been reported to localize at both cholinergic and non-cholinergic presynaptic terminals [108,109]. The M4 receptors are found at the presynaptic terminals of cholinergic interneurons within the striatum [110] and they also seem to be present in the medium spiny neurons of the direct pathway [111]. In contrast to the nicotinic cholinergic receptors, mAChRs act slower but exert potentially more sustained synaptic response acting through second messengers.

In schizophrenia, altered cholinergic neurotransmission is intimately linked to the defective cognitive functions associated primarily with cortical and hippocampal regions. Post-mortem studies consistently reported transcriptional and proteomic alterations in M_1_ and M_4_ receptors in the hippocampus [112,113] prefrontal and frontal cortices [112,114,115,116], and also cingulate cortex [117,118] of schizophrenic patients. Conversely, potentiation of the central muscarinic system by M_1_ mAChR’s positive allosteric modulator (PAM), completely restored defective long-term depression as well as impairments in the cognitive function and social interaction in PCP-treated mouse model of schizophrenia [119]. Interestingly, no significant differences in the density of M_2_ and M_3_ receptors between cortical regions of schizophrenic and control subjects have been observed [120]. It has been recently demonstrated that acetylcholinesterase inhibitors (AChEIs) or similar agents increasing ACh level may be effective in the treatment of visual hallucinations in individual clinical cases [121,122]. On the other side, the results of many clinical studies [123,124,125] did not show any improvement of schizophrenia symptoms by AChEIs or similar agents increasing ACh level. These results could suggest that contribution of the central muscarinic receptor system to schizophrenia deficits may not arise from disturbances in ACh level but rather involves far more complex changes underlying neuropathology of this disorder [126]. In non-psychotic individuals, administration of anti-muscarinic agents such as atropine or scopolamine evoked dose-dependent impairments in cognitive and psychomotor function including attention, learning process, working, and declarative memory [127,128,129].

Novel drugs targeting the allosteric binding site in mAChRs helped to extend our knowledge about the role of these receptors in Ca^2+^-dependent signal transduction in the brain and they turned out to be promising in the treatment of psychotic symptoms commonly observed in patients with schizophrenia. One of the modulators with pro-cognitive action is AC-260584, a potent agonist at the M_1_ receptor, that may mediate calcium responses and ERK1/2 activation in specific brain areas involved in learning and memory formation, such as the hippocampus, prefrontal and perirhinal cortex [130]. A previous report suggested that ACh may control the LTP induction in CA1 hippocampal pyramidal neurons by stimulating M_1_ receptor and leading to Ca^2+^ release from IP_3_-sensitive stores [131]. Moreover, the regulation of synaptic plasticity and cognitive function by muscarinic system can result from tuning the activity of non-glutamatergic postsynaptic ion channels including voltage- or Ca^2+^-gated channels [132,133]. Consistent with these findings, administration of 77-LH-28-1, another allosteric agonist of M_1_ receptor, led to M_1_ receptor-dependent inhibition of calcium-activated potassium (SK) channels, promoting the induction of NMDAR-dependent LTP [134]. The M_1_ receptors via signaling cascade linking cAMP-PKA and PI3K-Akt-mTOR may also be critical for the activation of postsynaptic AMPA receptors needed for the LTP [135,136].

The synaptic AMPA receptors and mTOR signaling pathways have been demonstrated to be significantly disrupted in schizophrenia [137,138]. The function of muscarinic system in the modulation of altered synaptic transmission may precipitate or exacerbate certain symptoms of psychiatric disorders. Interestingly, Jeon et al. revealed that muscarinic blockade of D_1_ receptor-induced cAMP production was abolished in striatal neurons of D1-M4-KO mice model underlining physiological relevance of M_4_ receptors in dopamine-dependent behaviors and representing another potential therapeutic target in the treatment of schizophrenia [139].

## 6. Serotonergic Receptors

Serotonin (5-hydroxytryptamine, 5-HT) is one of the most extensively studied neurotransmitters, acting through distinct G protein coupled receptors (GPCRs) and ligand-gated ion channels [140]. The last two decades of research described at least fifteen 5-HT receptors subtypes, which are grouped into seven families (5-HT_1_-5-HT_7_) [141] based on the specific biochemical signaling pathways, as presented in Table 1 [142]. All subtypes have a distinct expression pattern across the central nervous system (Table 1). In the human brain, almost all serotonin receptors subtypes are found, except for 5-HT_5b_, and they play an important role in the modulation of cognitive and behavioral functions [140].

The considerable evidence for alterations in serotonin level in schizophrenia comes from pharmacological data. D-lysergic acid diethylamide (LSD), which is structurally similar to serotonin, induces psychotomimetic effects in non-psychiatric controls [143]. Further investigations demonstrated that LSD causes hallucinations through its agonistic effect on the 5-HT_2A_ receptors subtype [144]. To support it, the group of González–Maeso demonstrated that 5-HT_2A_ knock-out mice were unsusceptible to the neuropsychological effects of serotonergic psychedelics [145,146].

The 5-HT_2A_ receptors are present in high density in brain regions which are implicated in the pathophysiology of schizophrenia and play a key role in cognition, perception, and emotion regulation [147]. A large number of studies points to alterations in frontal cortical 5-HT_2A_ receptor binding in schizophrenic patients and the reduction in receptor density in schizophrenic brains compared to healthy individuals [148]. Furthermore, a new generation of antipsychotic drugs act through serotonin receptor-based mechanism [149]. They exhibit low prevalence of side effects and the effectiveness against both positive and negative symptoms. Despite intensive studies, the molecular and neurochemical bases of atypical drugs action have long been a matter of debate. It has been postulated that a high 5-HT_2A_ vs. dopamine D2 receptor occupancy is characteristic for atypical drugs and majority of them including clozapine, olanzapine, risperidone, or ziprasidone are characterized by high affinity for 5-HT_2A_ receptors [150]. However, not 5-HT_2A_ receptor antagonism per se but a combined blockage of D2 and 5-HT_2A_ receptors is believed to confer the efficacy of a second-generation antipsychotics [151,152]. Indeed, the atypical antipsychotics are frequently characterized by their combined action for the antagonism of 5-HT_2A_ and D2 receptors [153]. Studies have shown that this treatment strategy can efficiently reduce the negative and cognitive symptoms as well as minimize the side effects [154]. Additionally, equilibrium between 5-HT_2A_ and D2 receptor occupancy is crucial for minimizing extrapyramidal symptoms and improving efficacy in a treatment-resistant schizophrenia [155,156]. These have been assessed by several studies showing the beneficial effects of antagonism of 5-HT_2A_ and D2 receptors, notably using single or saturating doses of haloperidol [152,157,158], and recently in rats chronically treated with haloperidol alone or in combination with MDL-100,907, a selective antagonist of 5-HT_2A_ receptor [159].

Several findings have also pointed to the biological significance of serotonin receptor 2A gene in schizophrenia, but the results are inconclusive. For example, Sern-Yih Cheah and coworkers showed three potential risk factors for schizophrenia: the down-regulated 5HT_2A_ mRNA levels in the PFC, hypermethylation of 5HT_2A_ promoter CpG sites (cg5, cg7 and cg10) and genetic correlation with 5HT_2A_ genotypes for rs6314 and rs6313 [147]. On the other hand, postmortem study on untreated schizophrenic patients demonstrated up-regulation of 5HT_2A_ receptor density in the PFC [160]. In addition to genetic variations in 5HT_2A_, environmental factors can be also associated with 5HT_2A_ gene expression. There are multiple lines of evidence to demonstrate that 5-HT_2A_ receptors and metabotropic glutamate type 2 (mGlu2) receptors interact with each other and form functional complexes in brain cortex [160,161,162]. It has been demonstrated that the density of 5-HT_2A_/mGluR2 complex in the cortex of schizophrenic individuals is dysregulated [154]. The functional role of these complexes has also been studied in animals. For instance, stimulation of cells expressing functional 5-HT_2A_/mGluR2 heterocomplexes with mGluR2 agonist activated Gq/11 proteins by the 5-HT_2A_ receptors and this activation was abolished in 5-HT_2A_ knockout mice [161]. The mGluR2 knockout mice were resistent to the behavioral effects of hallucinogenic drugs [163], which suggests that 5-HT_2A_/mGluR2 complex may be obligatory for neuropsychological responses to hallucinogens. The postmortem studies demonstrated upregulation of 5-HT_2A_ receptor and downregulation of mGluR2 receptor [160], a pattern that may predispose psychosis.

Moreover, postmortem and neuroimaging studies also support a role of serotonergic system in the pathophysiology of schizophrenia [164]. Yasuno and coworkers showed decreased 5-HT_1A_ receptor binding in the amygdala, which may underlie the affective components included in schizophrenia symptoms [165]. Moreover, it has been demonstrated that atypical antipsychotic drugs enhance dopamine release in the prefrontal cortex through postsynaptic 5-HT_1A_ activity [166]. This observation may be essential for choosing an optimal treatment strategy, in which negative symptoms and cognitive deficits in schizophrenia have been linked to decreased function of dopaminoceptive neurons.

The 5HT_2C_, 5HT_6_, and 5HT_7_ receptors are also considered as pharmacological targets in the treatment of psychosis and cognitive deficits in schizophrenia [167]. For instance, the interaction of clozapine with 5HT_6_ receptors improves cholinergic signaling and may be helpful in the treatment of neurocognitive defects [168]. The anatomical distribution of 5HT_7_ receptor subtype in the human brain together with the reduction of mRNA levels of this receptor in the prefrontal cortex of schizophrenic individuals as well as the genetic correlation between 5HT_7_ receptors and schizophrenia emphasize their role in the development of this disorder [169]. A growing body of evidence indicates that schizophrenia has a strong neurodevelopmental component [170,171]. Therefore, it is highly plausible that the disease can be influenced by 5HT_6_ and 5HT_7_ receptors or other GPCRs controlling key neurodevelopmental processes.

Furthermore, the results of multiple studies demonstrated an association between serotonin receptor polymorphism and disease susceptibility for schizophrenia. The T102C polymorphism of the 5-HT_2A_ receptor and the C759T polymorphism of 5HT_2C_ receptor have been positively associated with positive and negative symptom response [172,173]. All these findings highlight a crucial role of serotonergic neurotransmission in the pathophysiology of schizophrenia. However, further studies are needed to improve efficiency of antipsychotic drug that modulate the activity serotonin receptors.

## 7. Glutamate Metabotropic Receptors

**Figure 5:**
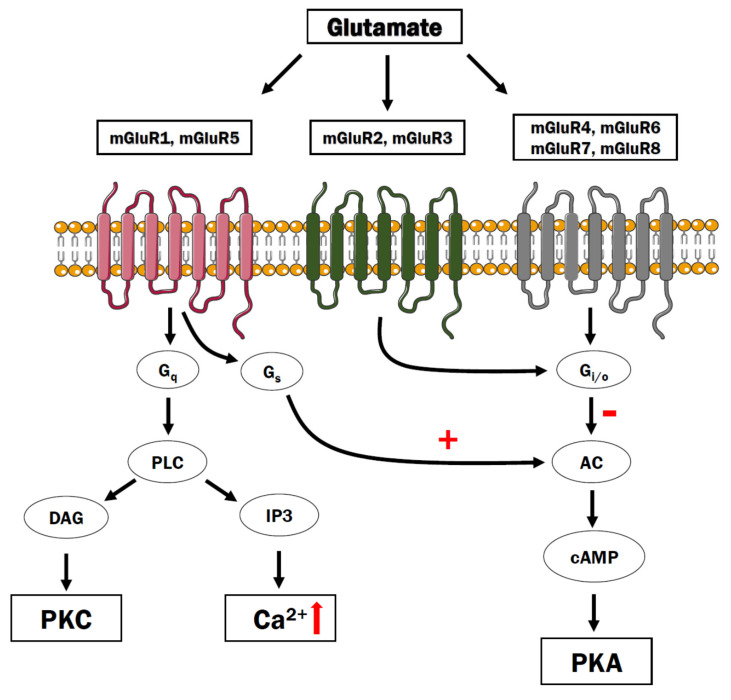
The metabotropic glutamate receptors. The group I mGluRs couples to Gq, which stimulates PLC activity and inositol 1,4,5-triphosphate (IP_3_) and diacylglycerol (DAG). The IP_3_ diffuses to the endoplasmic reticulum and activates the IP_3_ receptors to release Ca^2+^ to the cytosol. The Group I can also couple to adenylyl cyclase to stimulate cAMP production. By contrast, Groups II and III couple to G_i/o_ proteins and inhibit adenylyl cyclase.

Metabotropic glutamate receptors are encoded by GRM1 to GRM8 genes and have a modulatory function for the release of neurotransmitters, regulation of neuroplasticity, and synaptic excitability [174]. Based on receptor structure, ligand selectivity, and the psychological effect caused by activation of the receptor, mGluRs are classified into three groups: Group I, Group II, and Group III (Figure 5). Activation of Group I (mGluR1 and mGluR5) receptors causes phospholipase C-mediated effect, while Group II (mGluR2 and mGluR3) and Group III (mGluR4,6,7,8) receptors are associated with inhibition of cAMP signaling through G_i_/G_o_ protein [175]. All of the mGluR are present in neuron and glial cells, the only exception is mGluR6 which is primarily located in the retina [176].

The mGluR1 and mGluR5 belonging to Group I are located mainly in the postsynaptic site and act through phospholipase C-dependent Ca^2+^ mobilization and stimulation of adenylyl cyclase, albeit the contribution of other signaling pathways has been demonstrated as well [177,178]. In general terms, activation of these receptors leads to neuronal depolarization. However, the mGluR1 and mGluR5 can also modulate the pre- and postsynaptic current of the NMDA receptor in a Ca^2+^-dependent manner. An increase in Ca^2+^ level causes activation of mGluR1 and mGluR5, which results in decreased activity of the NMDA receptor and protection from detrimental consequences of Ca^2+^ overload [179]. So far, 12 rare mutations in the GRM1 gene were discovered and described as being correlated with disease etiology [180]. Moreover, postmortem studies demonstrated increased expression of mGluR1 in the prefrontal cortex in patients with schizophrenia [181]. A growing body of evidence indicates that both mGluR1 and mGluR5 should be considered as new molecular targets for schizophrenia treatment. Preclinical studies using PCP-, amphetamine (AMPH)-, or MK-801-induced animal models indicated that mGluR1′s or mGLuR5′s positive or negative allosteric modulators (PAMs or NAMs) can effectively reduce hyperlocomotion and ameliorate deficits in prepulse inhibition and social interactions [176,182,183]. For instance, mGluR5 agonist—VU0409551, produced rapid antipsychotic-like and cognition-enhancing activity in rodent models of schizophrenia and turned out to be effective in reversing the deficits in serine racemase knockout mice, a model that mimics many behavioral and neurochemical abnormalities observed in this disease [184].

The receptors from Group II are expressed only in a few brain regions: mGlu2 in the cerebellar and cerebral cortex, hippocampus, olfactory bulbs, and it is located in presynaptic, postsynaptic, or glial sites whereas mGlu3 is predominantly expressed in the dentate gyrus, nucleus accumbens, lateral septal nucleus, cerebral cortex, cerebellar cortex, striatum, substantia nigra pars reticulata, amygdaloid nuclei, and it is located only in the preterminal region of neurons away from synaptic sites [176,185]. Group II receptors act by inhibiting the adenylyl cyclase and voltage-dependent Ca^2+^ channels while activating voltage-dependent K^+^ channels [186]. Research on animal models of schizophrenia showed that pharmacological activation of mGluR2/3 decreased behavioral and cellular deficits of the NMDA receptor hypofunction and improved motor activity [187]. Numerous Group II mGluR’s agonists were checked for therapeutic efficacy in schizophrenia. In preclinical research, LY354740 improved working memory and caused stabilization in glutamatergic signaling in the PCP-induced model of NMDA receptor hypofunction [188]. In the same model, LY379268 decreased the deficits in prepulse inhibition and reduced the expression of falling, turning, and back pedaling in rats in a dose-dependent manner [189]. The studies with healthy volunteers showed that LY354740 produced significant dose-dependent improvement in working memory during ketamine challenge suggesting that mGluR2/3 may play a role in memory impairments related to NMDA receptor hypofunction [190]. Clinical trial with LY2140023, an oral prodrug of LY404039, demonstrated the improvement in both positive and negative symptoms of schizophrenia compared to placebo. LY2140023 was safe and well-tolerated, and patients did not face different from placebo extrapyramidal symptoms or weight gain [191]. As reviewed by Moreno and colleagues, mGluR2, but not mGluR3, is the receptor responsible for antipsychotic-like effects of mGluR2/3 agonists, at least in preclinical models. This is supported by the concurrent studies with LY404039 and LY379268 showing that the effects of mGluR2/3 agonists are abolished in mGluR2, but not in mGluR3, knockout mice [192]. Interestingly, mGluR2 PAMs have the effects comparable with mGluR2/3 orthosteric agonists as was shown for LY379268 and biphenyl-indanone A (BINA) in PCP- and AMPH-induced animal models [193,194].

The drugs targeting mGluR2/3 have also been tested in clinical trials. In the first run of randomized phase II, LY-2140023 initially improved both positive and negative, but not cognitive, symptoms of schizophrenia when compared to placebo but no differences were seen between tested and olanzapine positive group. The second trial showed no significant differences between LY-2140023 and olanzapine, risperidone, or aripiprazole groups over 6–8 weeks of treatment and further clinical investigations were ceased by the Eli Lilly company [191,195,196]. The mGluR2 PAM, ADX71149 showed safety, tolerance, and efficiency toward negative symptoms of schizophrenia in IIa phase of clinical trials. In a dose-dependent manner, it significantly ameliorated smoking withdrawal-evoked deficits in attention and episodic memory and reduced ketamine-evoked negative symptoms [197,198]. However, up to date no results of phase III have been released. In 2016, AstraZeneca disclosed the results of phase II of AZD8529, a selective mGluR2 PAM, but no significant improvement in negative and positive symptoms of schizophrenia was demonstrated [199]. Receptors of Group III mGluRs: mGluR4, mGluR6, mGluR7, mGluR8 are the least explored among all metabotropic glutamate receptors. They are located mainly in the presynaptic site of neurons with the exception of mGluR6, which is located in the postsynaptic site of bipolar retinal cells. Group III receptors are similar to Group II in terms of mechanism of action—they signal via Gα_i/o_ to inhibit adenyl cyclase and modulate the activity of other downstream effectors such as cGMP phosphodiesterase, MAPK, or PI3 kinase pathways [182,186,200]. It has been demonstrated that mGluR4 activation decreases glutamatergic transmission in the hippocampus [201] while mGluR4 knockout resulted in prepulse inhibition and lower acoustic startle response [202]. A variety of mGluR4 agonists were tested in preclinical studies. The LSP1-2111 was effective in reducing MK-801- and AMPH-induced hyperlocomotion and DOI (2,5-dimethoxy-4-iodoamphetamine)-induced head twitches [203]. The LSP4-2022 drug lowered neurotransmitter release caused by MK-801 and had an antipsychotic effect [204]. The LuAF21934 and LuAF32615 regulated hyperactivity induced by MK-801 and amphetamine and decreased head twitches caused by DOI [205]. Administration of the ADX88178 resulted in a reduction of hyperlocomotion caused by MK-801 and head twitches caused by DOI [206].

Knockout of mGluR7 in mice model worsened short-term neural plasticity in the hippocampus compared to the wild type, and produced deficits in memory and anxiety responses [207]. The mGluR7′s NAMs tested in preclinical studies—MMPIP and ADX71743—were successful in normalization of deficits caused by MK-801 and DOI-induced head twitches. However, ADX71743 needed lower doses to cause therapeutic effect compared to MMPIP [208,209]. Both drugs were also active when tested in models of cognition, attentional deficits, and social interactions [210]. Several other drugs targeting mGluR7 have been synthesized recently, for instance VU6010608 (2017) or VU6027459 (2020), but their utility in schizophrenia treatment has not been investigated yet.

Research on the role of mGluR8 in schizophrenia provided inconsistent results—some scientists demonstrated that knockout of this receptor resulted in subtle behavioral alterations including novelty-induced hyperactivity, delayed stimuli response [211] and anxiety [212]. However, these findings were not confirmed by others [213,214]. Similarly, some preclinical studies showed that mGluR8′s selective agonist (S)-3,4-dicarboxyphenylglycine (DCPG) decreased hyperactivity induced by pharmacological blockage of NMDA receptor while others did not confirm normalization of locomotor activity by the drug [215,216]. Despite these discrepancies, mGluR8 should still be considered as a potential molecular target in schizophrenia treatment.

## 8. GABAB Receptors

Gamma-aminobutyric acid (GABA) is the main inhibitory neurotransmitter in the brain. Many studies have demonstrated dysfunctions in GABA transmission in schizophrenia pathophysiology [217,218]. GABA activates fast synaptic inhibition via ionotropic GABAA receptors and slow synaptic inhibition via metabotropic GABAB receptors (GBRs) [219]. GBRs are G-protein coupled to K^+^/Ca^2+^ channels and consist of two closely related seventh transmembrane subunits—GABAB receptor 1 (GBR1) and GABAB receptor 2 (GBR2), both of them required to assembly into functional receptor. The GBR1 subtype exists in two splice variants—GABABR1a (130 kDa) and GABABR1b (100 kDa) [217]. GBR1 binds orthosteric ligands, while GBR2 couples with G protein [7], releasing Gα_i/o_ and G_βγ_ when activated [219]. In addition to GABA, GBRs activity can also modulate the release of dopamine and serotonin [220].

GBRs’ abundant expression in the cortex and their significant role in learning and memory formation indicate the importance of these receptors in the CNS, but the understanding of GBRs function is still limited [217,221].

A series of studies have reported abnormalities in GBRs in schizophrenia [218] and immunohistochemical experiments found decreased GBR1a immunolabeling in the hippocampus, prefrontal cortex, inferior temporal cortex, and the entorhinal cortex of schizophrenia patients [217,222]. In addition, the loci for both GABBR1 (6p21.3) and GABBR2 genes (5q34) have been recognized as the susceptibility loci for schizophrenia [218]. Fatemi and coworkers detected significant reduction in GABBR1 and GABBR2 protein level in the lateral cerebella and superior frontal cortex from patients with schizophrenia, bipolar disorder, and major depression when compared to healthy controls [218,220]. Though one report showed a weak correlation between GABBR1 gene and schizophrenia [223], two other found no connection [224,225]. In two microarray studies, increased expression of GABBR1 and GABBR2 mRNA was observed in the brain tissue from suicides [226]. Alterations in GBR subunits expression may disturb affinity, transmission, and receptor insertion into the plasma membrane, possibly promoting emotional and cognitive deficits in schizophrenia [220].

Despite the contribution of GBRs to schizophrenic symptoms and extensive drug discovery efforts, to date, only two GABAB receptor agonists—baclofen and gamma-hydroxybutyric acid (GHB)—have been introduced to the clinical use [227]. Baclofen has poor liposolubility and does not cross the blood–brain barrier (BBB) efficiently [227], but it systemic administration reduced behavioral hyperactivity and/or prepulse inhibition deficits in animal models of schizophrenic psychoses induced by methamphetamine [228], MK-801 [229], or phencyclidine [230]. Likewise, baclofen administered intraperitoneally reversed dizocilpine-induced prepulse inhibition disruption and spontaneous gating deficits in juvenile DBA/2 mice, and the effects were blocked by the pretreatment with a GBR antagonist [227]. In the prefrontal cortex and hippocampus of DBA/2 mice, decreased GBRs expression was found, suggesting that the schizophrenia-like phenotype may be connected to the disturbances in GABAergic system [227]. However, despite promising preclinical data, trials with baclofen on schizophrenic patients turned out disappointing. Other studies additionally demonstrated that baclofen could be responsible for hallucinations on severe withdrawal psychosis [217,220].

Second, GBRs agonist, GHB, has an advantage over baclofen in reaching significant CNS concentrations, due to the evidence for carrier-mediated transport across the BBB [227]. GHB may act directly as a neurotransmitter but also modulate dopamine transmission via the GHB receptor and GBRs after conversion to extracellular GABA [227]. Dopamine modulation seems to be regulated mainly by the GBR [231] since GABAB1 knockout mice do not display the same behavioral response to GHB administration as the wild-type [227].

The GBR antagonists and positive allosteric modulators (PAM) are under extensive studies due to their lack of undesirable side effects caused by baclofen [232]. Several preclinical investigations have demonstrated GBRs antagonists’ effectiveness in the treatment of cognitive dysfunctions in a rat model of absence epilepsy or improvement cognitive task performance by activating hippocampal θ and γ rhythms in behaving rats [227,233]. Other researchers demonstrated that GB receptor antagonist—SGS742—improved spatial memory, possibly due to a weaker binding to the cyclic adenosine monophosphate response element in the hippocampus [233]. Additionally, the infusion of GBRs antagonists—CGP56999A and CGP35348—into the rat hippocampus produced deficits in prepulse inhibition and affected hippocampal sensory and sensorimotor gating [234]. Another report on the animal model of schizophrenia—the apomorphine-susceptible (APO-SUS) rat and its phenotypic counterpart, the apomorphine-unsusceptible (APO-UNSUS) rat at postnatal day 20–22—showed that CGP55845 abolished prepulse inhibition reduction, suggesting that the diminished paired-pulse ratio was caused by increased GBRs signaling. Increased expression of the GB1 receptor subunit in APO-SUS rats seems to support it [235].

Research on schizophrenia animal models with positive allosteric modulators of GBRs showed that GS39783 blocked hyperlocomotion induced by MK-801 [236]. Similarly, CGP7930 co-administered with a low dose of baclofen reduced amphetamine-induced hyperlocomotion [237]. CGP7930 has also been described to antagonize psychosis-relevant behavior triggered by hippocampal kindling, including deficits of prepulse inhibition and gating of hippocampal auditory evoked potentials (AEPs) [234]. Furthermore, CGP7930 prevented ketamine—induced deficit of prepulse inhibition, suppressed hyperlocomotion, and reduced heterosynaptically mediated paired pulse depression in rat hippocampus [232]. The most recent analysis of the X-ray crystal structure of GBR suggests that clozapine—the gold-standard drug in the treatment of resistant schizophrenia—could directly bind to the GABAB receptor in a way similar to baclofen [238].

The signaling pathways downstream GBRs are related with one of three effector proteins: the GIRK family-G protein-activated inwardly rectifying K^+^ channels, voltage-gated N-type Ca^2+^ channels, and adenylyl cyclase [219,238]. The GB receptors interact with a variety of other signaling pathways, but these connections are not fully resolved yet. However, recent study revealed a new functional relationship between widely distributed GBRs and densely expressed sodium-activated potassium channels in the olfactory bulb neurons. Li and coworkers demonstrated a novel mechanism by which GBR activation inhibits two opposing currents, the persistent sodium current and the sodium-activated potassium current [221]. Broad colocalization of GBRs and sodium-activated potassium channels in the nervous system indicate an important mechanism for GBRs neuromodulation. These results suggest a new possibility for controlling cell excitability through GBRs modulators [221].

GBRs control synaptic transmission by either inhibiting neurotransmitter release or diminishing postsynaptic excitability. Presynaptic GBRs inhibit neurotransmitter release by modulating calcium channels or interacting with the downstream release machinery [219]. GBRs dampen postsynaptic excitability by releasing G_βγ_ subunits to activate inwardly rectifying K^+^ channels. Local shunting and slow inhibitory postsynaptic potentials (IPSPs) generated by opening of these channels, enhance magnesium blockage of NMDARs and indirectly inhibit synaptic responses [239]. This indirect blockage of NMDARs together with inhibition of voltage-sensitive Ca^2+^ channels indicate a significant mechanism by which GBRs influence calcium signaling in dendrites and spines [240]. Especially, postsynaptic GBRs are frequently located in and near dendritic spines, making them well-positioned to influence glutamate receptors [219,241].

Consistently, two-photon optical quantal analysis revealed that presynaptic GBRs suppress multivesicular release at individual synapses from layer 2/3 pyramidal neurons in the mouse medial prefrontal cortex [219]. The same authors also showed that postsynaptic GBRs directly modulate NMDARs via the PKA pathway. These results demonstrated a new role for postsynaptic GBRs directly suppressing NMDAR Ca^2+^ signals, with little impact on AMPAR or NMDAR synaptic currents [219]. This potent GBRs modulation depends on G protein signaling and involves PKA pathway. Direct suppression of NMDAR calcium signals by GBRs suggests that GBRs have an ability to modulate not only electrical properties of neurons, but also to influence biochemical signaling cascades at the synapses. This is an important mechanism by which GABA signaling helps to control neuronal communication in the brain [219].

## 9. Chemokine Receptors

### 9.1. Classification of Chemokines and Their Receptors

**Figure 6:**
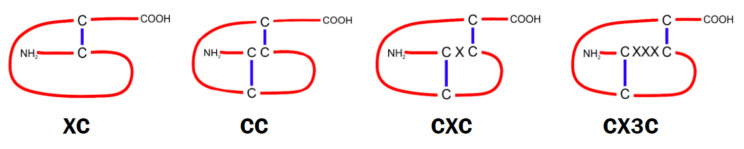
Schematic structures of chemokine subtypes.

Chemotactic cytokines (chemokines) are small alkaline peptides (7 to 15 kDa) known as the important mediators of inflammatory processes. Based on the number of amino acids between the two cysteines at the amine end of the molecule, they are classified into four groups: XC, CC, CXC, and CX3C, where C is cysteine and X represents another amino acid [242]. Chemokines usually possess the conserved four cysteines and formation of disulfide bridges determines their three-dimensional structure (Figure 6). So far, about 50 chemokines and 10 receptors for CC subtype, 7 for CXC subtype, and single receptors for XC and CX3C chemokines have been identified [243]. Although most chemokine receptors belong to the classic G protein receptors, there is also a group of so-called atypical chemokine receptors (ACKRs), with at least 6 representatives [244]. They bind chemokines with high affinity, but due to their structural inability to couple to G proteins, they do not induce cell migration and act mainly as “capturers” of chemokine, reducing inflammation or shaping chemokine gradients [245]. Signals from chemokine receptors are transmitted by two major routes: G proteins and β-arrestin; however, these processes are cell- and tissue-dependent, and can be modulated by the ligands or receptors involved [246,247].

A particularly important aspect of chemokine-induced signaling is that chemokine receptors could bind several chemokines as well as can act as multimeric forms, homo- or heterodimers [248,249,250]. Moreover, some chemokines can form complexes with more than one receptor, thereby overlapping mechanisms may differentiate the final biological effect. Crucial is the affinity of a given chemokine to the receptor and the density of particular receptor types in the cell. Recently, the phenomenon of chemokine receptor oligomerization, which specifically modifies the response to chemokine binding, has become increasingly important. Composition of homo- or heterooligomeric complexes determines their affinity for chemokine, which can lead to the activation of different signaling pathways [251]. In addition, signaling biases have been documented for several chemokine GPCRs [243,252].

Functionally, chemokines and their receptors play an important role in the nervous system, acting as trophic and protective factors that increase neuronal survival, regulate neuronal migration, and synaptic transmission. Chemokines can be classified as inflammatory or homeostatic, according to the context of their functioning [249]. They are constantly secreted and are responsible for proper cell migration, e.g., during the growth of the body. In the brain, the level of chemokines increases due to their secretion by many different cells: microglia, astrocytes, oligodendrocytes, and endothelial cells of blood vessels [253,254]. A particular role is played by the BBB and the most important is the precise exchange of chemical compounds between the CNS and the circulatory system [255]. The integrity of the BBB structure sustains brain homeostasis and allows to perform many neurological functions. Chemokines play a special role primarily in some CNS diseases, when the damages to the BBB and the blood–spinal/cerebral fluid barrier cause leukocytes infiltration triggering inflammatory processes [256].

### 9.2. Chemokines and Their Receptors in Schizophrenia

Although chemokines can trigger a number of downstream signaling pathways, we focused on those involving PLC activity, because of the significance of Ca^2+^ released from the endoplasmic reticulum. Among all chemokine subtypes, only several are known to play a role in schizophrenia, but due to limited and sometimes conflicted data, their participation should be analyzed with caution. The discrepancies could result from heterogeneity of examined group of schizophrenic patients, including duration of the disease, age, sex, and treatment response [257,258]. Chemokine levels are mainly determined in the serum, but since their receptors’ expression may vary in different cells, the chemokine concentration does not always correlate with schizophrenic symptoms. It may complicate the explanation of the role played by particular chemokines in schizophrenic insults. However, based on many studies performed recently, with the pro-inflammatory action at least a few chemokines appears to be strongly associated with the disease state. The activation of PLC-sensitive signaling pathways has been demonstrated for many chemokines with the prevalence of those belonging to CC and CXC classes and one representative of CX3C type (Table 2).

The elevations of inflammatory chemokines in blood and cerebrospinal fluid as well as altered function of immune cells in the central nervous system deregulate the chemokine-mediated network that may contribute to the progression of schizophrenia [259,260]. The processes triggered by migration of immune cells to the brain may also impair neuron–microglia crosstalk by hyperactivation of astrocytes and microglial cells. Subsequent release of pro-inflammatory chemokines activates chemokine receptors followed by the raise in cytosolic Ca^2+^, affects chemotaxis, secretion, and gene expression. The chemokines can be also released by activated astrocytes, thus inducing production of reactive oxygen species (ROS) leading to excitotoxic neuronal death. Nowadays, inflammation constitutes an apparent risk factor for schizophrenia and increased chemokines production during inflammatory conditions may play a role in development of the disease. Noteworthy, chemokines can be rapidly transported from the blood to the brain through the BBB and trigger a cascade of events contributing to alterations in BBB integrity and development of BBB breakdown [256].

Accumulating evidence indicates that increased level of pro-inflammatory chemokines: CCL2, CCL4, CCL11, CCL17, CCL22, and CCL24, in serum strongly correlates with schizophrenic symptoms including cognitive impairments in attention, working memory, episodic and semantic memory, and executive functions [11,261,262,263,264,265,266,267,268]. Several chemokines of CXC type (CXCL8, CXCL11, CXCL12), were also shown to act through PLC/Ca^2+^ downstream signaling [269,270,271]. An interesting observation was that several prenatal infections and inflammatory biomarkers may contribute to the etiology of schizophrenia, including fetal exposure to CXCL8 that could alter early stages of neurodevelopment [272,273].

Circulating chemokines detectable in serum may be produced by blood cells, endothelium or may originate from the brain. Hence, their concentration determined in situ may not always reflect their tissue levels. Moreover, due to different chemokines’ half-life and higher concentration at the sites of release, the concentration determined in the blood may not correlate with the physiological response. Therefore, the chemokine nature appears to be ambivalent: they can be protective or contribute to neuronal damage. The obligatory element to initiate chemokine signal transmission is the presence of responsive receptors.

**Figure 7:**
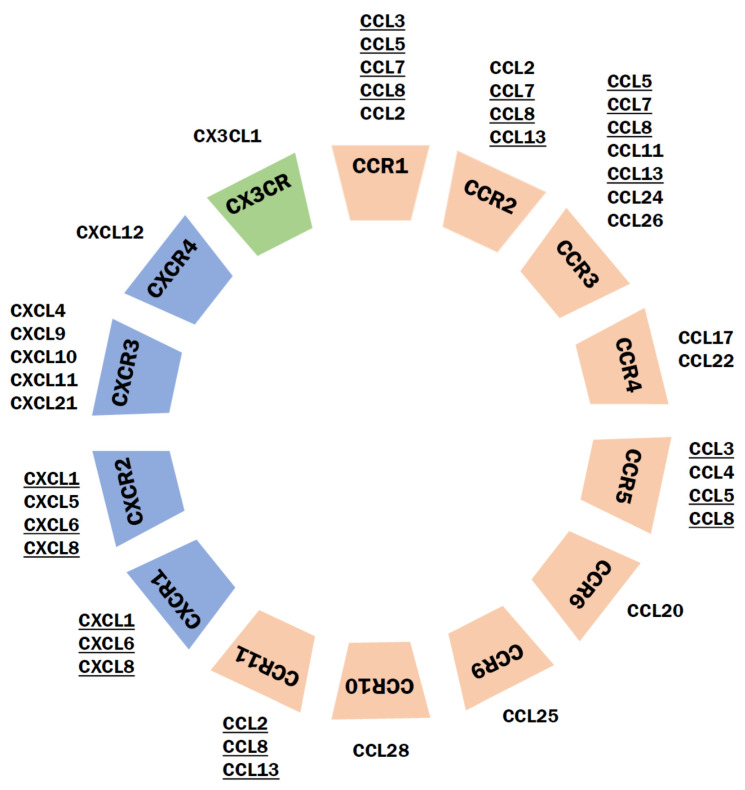
Chemokines and their receptors in schizophrenia. Some chemokines (underlined here) that are altered in schizophrenia can bind to multiple receptors and they all act through increasing calcium transients. Based on [10,11,261,264,267,268,269,275,281,282,283,284,285,286,287,288,289,290,291,292,293].

Whereas the analysis of chemokines’ level in schizophrenic patients is quite complex, less information is available for chemokine receptors. As shown in Figure 7, a large number of chemokine receptors can bind more than one ligand. Moreover, the receptors can be differentially expressed in the CNS, making the separation of causes from the effects even more complicated. For example, there is an evidence that CCL-11 at low concentrations can act as a partial agonist at CCR2 and antagonize CCL2 activity, but high concentrations are sufficient to activate CCR2 in chemotaxis assays [261,274]. Some receptors have been proposed to form putative heteroreceptor complexes with an NMDA receptor (NMDAR-CCR2, NMDAR-CXCR4) that may also contribute to schizophrenia-like symptoms in mild neuroinflammation [275]. The function of CX3CR1 seems to be the most characterized since this receptor binds single chemokine—CX3CL1, which is the only chemokine with the expression higher in the CNS than in the periphery [276,277]. Communication of microglia with neurons via CX3CR1 signaling is involved in the formation of dendritic spines, facilitates neuron–microglia interactions, influences microglial activation and synaptic function [278]. Moreover, CX3CL1/CX3CR1 signaling regulates activation of microglia in response to brain injury or inflammation, and induces the response that may have either beneficial or detrimental effects [279,280]. An additional issue is that some chemokines may bind to the receptors without inducing transmembrane signals. Thus, even if the concentration of chemokines in blood of schizophrenic patients is increased, no evident changes at a physiological and behavioral level could be detected [11,267,281]. This phenomenon may, at least in part, explain the contradictory results reported in several studies.

Numerous mechanisms have been discovered in terms of activity regulation of chemokines and their receptors [250]. Available data demonstrate that around 40% of the schizophrenic patients have some degree of inflammation engaging chemokine/receptor complexes [294,295]. In addition, increased permeability of the BBB in a subset of patients with schizophrenia correlates with enhanced chemokine signaling [256]. Despite the progress that has been made regarding the role of chemokines and inflammatory processes in schizophrenia pathology, the available data are still sparse and mostly correlative. Moreover, inflammation has been detected in numerous neuropsychiatric diseases, thus limiting its relevance to discoveries of new therapeutic approaches of schizophrenia.

## 10. Concluding Remarks

An increasing number of reports on schizophrenia clearly indicates its multifactorial etiology, including genomic, epigenetic, endocrinological, and environmental components, which act synergistically to produce disease-specific symptomology. As we reviewed here, there is also a considerable body of evidence to support abnormalities in neurotransmitter-GPCRs signaling as an integral piece of schizophrenia neurobiology. The pathophysiology of this illness involves profound changes in motoric function, mood, and cognition derived from dysfunctional limbic system. The monoamine and neuropeptide pathways have been demonstrated to originate and project within hippocampus, thalamus, or brainstem. Therefore, it is not surprising that abnormalities in neurotransmitter systems are in the center of both preclinical and clinical studies. However, the existence of potential alterations in GPCR signaling suggests that the relief for patients resistant to current medications will be possible only by targeting post-receptor sites. Growing body of evidence indicates that the mechanisms underlying the synthesis and inactivation of second messengers may also offer the promise for the rational design and development of efficient drugs for schizophrenia treatment. Moreover, as the signal transduction pathways downstream GPCRs frequently display unique characteristics, they offer unique targets for relative specificity of action and hold much promise for novel drugs in the long-term schizophrenia treatment. However, the difficulty to transform preclinical results into clinically efficient treatment strategies is invariably the biggest challenge for the next era in neuropsychopharmacology.

## Figures and Tables

**Table 1:** The family of 5-HT receptors. Prepared based on [140,142].

Receptor	Potential	Type	Mechanism of Action	CNS Distribution
5-HT_1_ (5-HT_1A, 1B, 1D–F_)	Inhibitory	G_i_/G_0_-protein coupled	Inhibition of AC and decreasing intracellular concentration of cAMP	cerebral and frontal cortex, hippocampus, striatum, olfactory bulb, substantia nigra
5-HT_2_ (5-HT_2A–C_)	Excitatory	G_q11_- protein coupled	Activation of PLC, increasing intracellular concentration of IP3 and DAG, and increasing intracellular calcium	nucleus accumbens, basal ganglia, cerebellum, hypothalamus
5-HT_3_ (5-HT_3A,3B_)	Excitatory	Ligand-gated Na+/K+ channel	Depolarization of cell plasma membrane	hippocampus, amygdala, nucleus accumbens
5-HT_4_ (5-HT_4A–H_)	Excitatory	G_s_-protein coupled	Activation of AC and increasing intracellular concentration of cAMP	hippocampal membranes
5-HT_5_ (5-HT_5A_)	Inhibitory	G_i_/G_0_-protein coupled	Inhibition of AC and decreasing intracellular concentration of cAMP	olfactory bulb, neocortex, hippocampus, caudate putamen
5-HT_6_	Excitatory	G_s_-protein coupled	Activation of AC and increasing intracellular concentration of cAMP	thalamus, hypothalamus, hippocampus
5-HT_7_ (5-HT_7A–D_)	Excitatory	G_s_-protein coupled	Activation of AC and increasing intracellular concentration of cAMP	thalamus, hypothalamus, hippocampus

**Table 2:** Chemokines identified in schizophrenia (new and most popular old nomenclature).

CCL2	CCL3	CCL4	CCL5	CCL7	CCL8	CCL11	CCL13	CCL17	CCL20
MCP-1	MIP-1α	MIP-1β	RANTES	MCP-3	MCP-2	Eotaxin-1	MCP-4	TARC	MIP-3α
CCL22	CCL23	CCL24	CCL25	CCL26	CCL28				
ABCD-1	MPIF-1	MPIF-2	TECK	MIP-4α	MEC				
		Eotaxin-2		Eotaxin-3					
CXCL1	CXCL4	CXCL5	CXCL6	CXCL8	CXCL9	CXCL10	CXCL11	CXCL12	CXCL21
MGSA	PF4	ENA78	GCP-2	IL-8	MIG	IP-10	I-TAC	SDF1	SLC
CX3CL1									
Fractalkine									

## Data Availability

No new data were created or analyzed in this study. Data sharing is not applicable to this paper.

## References

[1] Charlson F.J., Ferrari A.J., Santomauro D.F., Diminic S., Stockings E., Scott J.G., McGrath J.J., Whiteford H.A. (2018). Global Epidemiology and Burden of Schizophrenia: Findings From the Global Burden of Disease Study 2016. Schizophr. Bull..

[2] Bowie C.R., Harvey P.D. (2006). Cognitive deficits and functional outcome in schizophrenia. Neuropsychiatr. Dis. Treat..

[3] Correll C.U., Schooler N.R. (2020). Negative Symptoms in Schizophrenia: A Review and Clinical Guide for Recognition, Assessment, and Treatment. Neuropsychiatr. Dis. Treat..

[4] Galderisi S., Mucci A., Buchanan R.W., Arango C. (2018). Negative symptoms of schizophrenia: New developments and unanswered research questions. Lancet Psychiatry.

[5] Toda M., Abi-Dargham A. (2007). Dopamine hypothesis of schizophrenia: Making sense of it all. Curr. Psychiatry Rep..

[6] Seeman P. (2013). Schizophrenia and dopamine receptors. Eur. Neuropsychopharmacol..

[7] Iasevoli F., Tomasetti C., Buonaguro E.F., de Bartolomeis A. (2014). The glutamatergic aspects of schizophrenia molecular pathophysiology: Role of the postsynaptic density, and implications for treatment. Curr. Neuropharmacol..

[8] Stefansson H., Rujescu D., Cichon S., Pietiläinen O.P.H., Ingason A., Steinberg S., Fossdal R., Sigurdsson E., Sigmundsson T., Buizer-Voskamp J.E. (2008). Large recurrent microdeletions associated with schizophrenia. Nature.

[9] Dickerson F., Stallings C., Origoni A., Schroeder J., Katsafanas E., Schweinfurth L., Savage C., Khushalani S., Yolken R. (2016). Inflammatory Markers in Recent Onset Psychosis and Chronic Schizophrenia. Schizophr. Bull..

[10] Stuart M.J., Baune B.T. (2014). Chemokines and chemokine receptors in mood disorders, schizophrenia, and cognitive impairment: A systematic review of biomarker studies. Neurosci. Biobehav. Rev..

[11] Hong S., Lee E.E., Martin A.S., Soontornniyomkij B., Soontornniyomkij V., Achim C.L., Reuter C., Irwin M.R., Eyler L.T., Jeste D.V. (2017). Abnormalities in chemokine levels in schizophrenia and their clinical correlates. Schizophr. Res..

[12] Réaux-Le Goazigo A., Van Steenwinckel J., Rostène W., Mélik Parsadaniantz S. (2013). Current status of chemokines in the adult CNS. Prog. Neurobiol..

[13] Grundmann M., Merten N., Malfacini D., Inoue A., Preis P., Simon K., Rüttiger N., Ziegler N., Benkel T., Schmitt N.K. (2018). Lack of beta-arrestin signaling in the absence of active G proteins. Nat. Commun..

[14] O’Hayre M., Eichel K., Avino S., Zhao X., Steffen D.J., Feng X., Kawakami K., Aoki J., Messer K., Sunahara R. (2017). Genetic evidence that β-arrestins are dispensable for the initiation of β. Sci. Signal..

[15] Komatsu H., Fukuchi M., Habata Y. (2019). Potential Utility of Biased GPCR Signaling for Treatment of Psychiatric Disorders. Int. J. Mol. Sci..

[16] Stępnicki P., Kondej M., Kaczor A.A. (2018). Current Concepts and Treatments of Schizophrenia. Molecules.

[17] Neves S.R., Ram P.T., Iyengar R. (2002). G protein pathways. Science.

[18] Khan S.M., Sung J.Y., Hébert T.E. (2016). Gβγ subunits-Different spaces, different faces. Pharmacol. Res..

[19] Bologna Z., Teoh J.P., Bayoumi A.S., Tang Y., Kim I.M. (2017). Biased G Protein-Coupled Receptor Signaling: New Player in Modulating Physiology and Pathology. Biomol. Ther. (Seoul).

[20] Bergmeier W., Weidinger C., Zee I., Feske S. (2013). Emerging roles of store-operated Ca^2+^ entry through STIM and ORAI proteins in immunity, hemostasis and cancer. Channels (Austin).

[21] Villalobo A., Ishida H., Vogel H.J., Berchtold M.W. (2018). Calmodulin as a protein linker and a regulator of adaptor/scaffold proteins. Biochim. Biophys. Acta Mol. Cell Res..

[22] Jung H., Miller R.J. (2008). Activation of the nuclear factor of activated T-cells (NFAT) mediates upregulation of CCR2 chemokine receptors in dorsal root ganglion (DRG) neurons: A possible mechanism for activity-dependent transcription in DRG neurons in association with neuropathic pain. Mol. Cell. Neurosci..

[23] Lee J.U., Kim L.K., Choi J.M. (2018). Revisiting the Concept of Targeting NFAT to Control T Cell Immunity and Autoimmune Diseases. Front. Immunol..

[24] Al Akoum C., Akl I., Rouas R., Fayyad-Kazan M., Falha L., Renno T., Burny A., Lewalle P., Fayyad-Kazan H., Badran B. (2015). NFAT-1, Sp-1, Sp-3, and miR-21: New regulators of chemokine C receptor 7 expression in mature human dendritic cells. Hum. Immunol..

[25] Liu M., Zhang S.B., Luo Y.X., Yang Y.L., Zhang X.Z., Li B., Meng Y., Chen Y.J., Guo R.X., Xiong Y.C. (2020). NFATc2-dependent epigenetic upregulation of CXCL14 is involved in the development of neuropathic pain induced by paclitaxel. J. Neuroinflamm..

[26] Fernandez A.M., Fernandez S., Carrero P., Garcia-Garcia M., Torres-Aleman I. (2007). Calcineurin in reactive astrocytes plays a key role in the interplay between proinflammatory and anti-inflammatory signals. J. Neurosci..

[27] Flatow J., Buckley P., Miller B.J. (2013). Meta-analysis of oxidative stress in schizophrenia. Biol. Psychiatry.

[28] Gonzalez-Liencres C., Tas C., Brown E.C., Erdin S., Onur E., Cubukcoglu Z., Aydemir O., Esen-Danaci A., Brüne M. (2014). Oxidative stress in schizophrenia: A case-control study on the effects on social cognition and neurocognition. BMC Psychiatry.

[29] Madireddy S. (2020). Regulation of Reactive Oxygen Species-Mediated Damage in the Pathogenesis of Schizophrenia. Brain Sci..

[30] Upthegrove R., Khandaker G.M. (2020). Cytokines, Oxidative Stress and Cellular Markers of Inflammation in Schizophrenia. Curr. Top. Behav. Neurosci..

[31] Ermakov E.A., Dmitrieva E.M., Parshukova D.A., Kazantseva D.V., Vasilieva A.R., Smirnova L.P. (2021). Oxidative Stress-Related Mechanisms in Schizophrenia Pathogenesis and New Treatment Perspectives. Oxid. Med. Cell. Longev..

[32] Bernstein H.G., Keilhoff G., Steiner J., Dobrowolny H., Bogerts B. (2011). Nitric oxide and schizophrenia: Present knowledge and emerging concepts of therapy. CNS Neurol. Disord. Drug Targets.

[33] Nasyrova R.F., Ivashchenko D.V., Ivanov M.V., Neznanov N.G. (2015). Role of nitric oxide and related molecules in schizophrenia pathogenesis: Biochemical, genetic and clinical aspects. Front. Physiol..

[34] Bitanihirwe B.K., Woo T.U. (2011). Oxidative stress in schizophrenia: An integrated approach. Neurosci. Biobehav. Rev..

[35] Morris G., Walker A.J., Walder K., Berk M., Marx W., Carvalho A.F., Maes M., Puri B.K. (2021). Increasing Nrf2 Activity as a Treatment Approach in Neuropsychiatry. Mol. Neurobiol..

[36] Boll K.M., Noto C., Bonifácio K.L., Bortolasci C.C., Gadelha A., Bressan R.A., Barbosa D.S., Maes M., Moreira E.G. (2017). Oxidative and nitrosative stress biomarkers in chronic schizophrenia. Psychiatry Res..

[37] Rajasekaran A., Venkatasubramanian G., Berk M., Debnath M. (2015). Mitochondrial dysfunction in schizophrenia: Pathways, mechanisms and implications. Neurosci. Biobehav. Rev..

[38] Flippo K.H., Strack S. (2017). An emerging role for mitochondrial dynamics in schizophrenia. Schizophr. Res..

[39] Baumeister A.A., Francis J.L. (2002). Historical development of the dopamine hypothesis of schizophrenia. J. Hist. Neurosci..

[40] Missale C., Nash S.R., Robinson S.W., Jaber M., Caron M.G. (1998). Dopamine receptors: From structure to function. Physiol. Rev..

[41] Sidhu A., Niznik H.B. (2000). Coupling of dopamine receptor subtypes to multiple and diverse G proteins. Int. J. Dev. Neurosci..

[42] Neve K.A., Seamans J.K., Trantham-Davidson H. (2004). Dopamine receptor signaling. J. Recept. Signal Transduct. Res..

[43] Svenningsson P., Nishi A., Fisone G., Girault J.A., Nairn A.C., Greengard P. (2004). DARPP-32: An integrator of neurotransmission. Annu. Rev. Pharmacol. Toxicol..

[44] Albert K.A., Hemmings H.C., Adamo A.I., Potkin S.G., Akbarian S., Sandman C.A., Cotman C.W., Bunney W.E., Greengard P. (2002). Evidence for decreased DARPP-32 in the prefrontal cortex of patients with schizophrenia. Arch. Gen. Psychiatry.

[45] Kosaka J., Takahashi H., Ito H., Takano A., Fujimura Y., Matsumoto R., Nozaki S., Yasuno F., Okubo Y., Kishimoto T. (2010). Decreased binding of [11C]NNC112 and [11C]SCH23390 in patients with chronic schizophrenia. Life Sci..

[46] Takahashi H., Kato M., Takano H., Arakawa R., Okumura M., Otsuka T., Kodaka F., Hayashi M., Okubo Y., Ito H. (2008). Differential contributions of prefrontal and hippocampal dopamine D(1) and D(2) receptors in human cognitive functions. J. Neurosci..

[47] Domyo T., Kurumaji A., Toru M. (2001). An increase in [3H]SCH23390 binding in the cerebral cortex of postmortem brains of chronic schizophrenics. J. Neural Transm. (Vienna).

[48] Felsing D.E., Jain M.K., Allen J.A. (2019). Advances in Dopamine D1 Receptor Ligands for Neurotherapeutics. Curr. Top. Med. Chem..

[49] Saucedo Uribe E., Carranza Navarro F., Guerrero Medrano A.F., García Cervantes K.I., Álvarez Villalobos N.A., Acuña Rocha V.D., Méndez Hernández M., Millán Alanís J.M., Hinojosa Cavada C.M., Zúñiga Hernández J.A. (2020). Preliminary efficacy and tolerability profiles of first versus second-generation Long-Acting Injectable Antipsychotics in schizophrenia: A systematic review and meta-analysis. J. Psychiatr. Res..

[50] Lee E.S., Kronsberg H., Findling R.L. (2020). Psychopharmacologic Treatment of Schizophrenia in Adolescents and Children. Child Adolesc. Psychiatr. Clin. N. Am..

[51] Muench J., Hamer A.M. (2010). Adverse effects of antipsychotic medications. Am. Fam. Physician.

[52] Ishigooka J., Iwashita S., Tadori Y. (2018). Efficacy and safety of brexpiprazole for the treatment of acute schizophrenia in Japan: A 6-week, randomized, double-blind, placebo-controlled study. Psychiatry Clin. Neurosci..

[53] Amada N., Akazawa H., Ohgi Y., Maeda K., Sugino H., Kurahashi N., Kikuchi T., Futamura T. (2019). Brexpiprazole has a low risk of dopamine D. Neuropsychopharmacol Rep..

[54] Vyas P., Hwang B.J., Brašić J.R. (2020). An evaluation of lumateperone tosylate for the treatment of schizophrenia. Expert Opin. Pharmacother..

[55] Li Y.C., Kellendonk C., Simpson E.H., Kandel E.R., Gao W.J. (2011). D2 receptor overexpression in the striatum leads to a deficit in inhibitory transmission and dopamine sensitivity in mouse prefrontal cortex. Proc. Natl. Acad. Sci. USA.

[56] Petty A., Cui X., Tesiram Y., Kirik D., Howes O., Eyles D. (2019). Enhanced Dopamine in Prodromal Schizophrenia (EDiPS): A new animal model of relevance to schizophrenia. NPJ Schizophr..

[57] Abi-Dargham A. (2004). Do we still believe in the dopamine hypothesis? New data bring new evidence. Int. J. Neuropsychopharmacol..

[58] Gross G., Drescher K. (2012). The role of dopamine D(3) receptors in antipsychotic activity and cognitive functions. Handb. Exp. Pharmacol..

[59] Watson D.J., Loiseau F., Ingallinesi M., Millan M.J., Marsden C.A., Fone K.C. (2012). Selective blockade of dopamine D3 receptors enhances while D2 receptor antagonism impairs social novelty discrimination and novel object recognition in rats: A key role for the prefrontal cortex. Neuropsychopharmacology.

[60] Lane H.Y., Lee C.C., Chang Y.C., Lu C.T., Huang C.H., Chang W.H. (2004). Effects of dopamine D2 receptor Ser311Cys polymorphism and clinical factors on risperidone efficacy for positive and negative symptoms and social function. Int. J. Neuropsychopharmacol..

[61] Staddon S., Arranz M.J., Mancama D., Perez-Nievas F., Arrizabalaga I., Anney R., Buckland P., Elkin A., Osborne S., Munro J. (2005). Association between dopamine D3 receptor gene polymorphisms and schizophrenia in an isolate population. Schizophr. Res..

[62] Malhotra A.K., Goldman D., Buchanan R.W., Rooney W., Clifton A., Kosmidis M.H., Breier A., Pickar D. (1998). The dopamine D3 receptor (DRD3) Ser9Gly polymorphism and schizophrenia: A haplotype relative risk study and association with clozapine response. Mol. Psychiatry.

[63] Kapur S., Seeman P. (2002). NMDA receptor antagonists ketamine and PCP have direct effects on the dopamine D(2) and serotonin 5-HT(2)receptors-implications for models of schizophrenia. Mol. Psychiatry.

[64] Lindefors N., Barati S., O’Connor W.T. (1997). Differential effects of single and repeated ketamine administration on dopamine, serotonin and GABA transmission in rat medial prefrontal cortex. Brain Res..

[65] Moghaddam B., Adams B., Verma A., Daly D. (1997). Activation of glutamatergic neurotransmission by ketamine: A novel step in the pathway from NMDA receptor blockade to dopaminergic and cognitive disruptions associated with the prefrontal cortex. J. Neurosci..

[66] Can A., Zanos P., Moaddel R., Kang H.J., Dossou K.S., Wainer I.W., Cheer J.F., Frost D.O., Huang X.P., Gould T.D. (2016). Effects of Ketamine and Ketamine Metabolites on Evoked Striatal Dopamine Release, Dopamine Receptors, and Monoamine Transporters. J. Pharmacol. Exp. Ther..

[67] Lisman J.E., Coyle J.T., Green R.W., Javitt D.C., Benes F.M., Heckers S., Grace A.A. (2008). Circuit-based framework for understanding neurotransmitter and risk gene interactions in schizophrenia. Trends Neurosci..

[68] Bergson C., Levenson R., Goldman-Rakic P.S., Lidow M.S. (2003). Dopamine receptor-interacting proteins: The Ca(2+) connection in dopamine signaling. Trends Pharmacol. Sci..

[69] Kabbani N., Woll M.P., Nordman J.C., Levenson R. (2012). Dopamine receptor interacting proteins: Targeting neuronal calcium sensor-1/D2 dopamine receptor interaction for antipsychotic drug development. Curr. Drug Targets.

[70] O’Donnell J., Zeppenfeld D., McConnell E., Pena S., Nedergaard M. (2012). Norepinephrine: A neuromodulator that boosts the function of multiple cell types to optimize CNS performance. Neurochem. Res..

[71] Saboory E., Ghasemi M., Mehranfard N. (2020). Norepinephrine, neurodevelopment and behavior. Neurochem. Int..

[72] Vasudevan N.T., Mohan M.L., Goswami S.K., Naga Prasad S.V. (2011). Regulation of β-adrenergic receptor function: An emphasis on receptor resensitization. Cell Cycle.

[73] Brueckner F., Piscitelli C.L., Tsai C.J., Standfuss J., Deupi X., Schertler G.F. (2013). Structure of β-adrenergic receptors. Methods Enzymol..

[74] Zheng J., Shen H., Xiong Y., Yang X., He J. (2010). The beta1-adrenergic receptor mediates extracellular signal-regulated kinase activation via Galphas. Amino Acids.

[75] Knaus A.E., Muthig V., Schickinger S., Moura E., Beetz N., Gilsbach R., Hein L. (2007). Alpha2-adrenoceptor subtypes--unexpected functions for receptors and ligands derived from gene-targeted mouse models. Neurochem. Int..

[76] Ramos B.P., Arnsten A.F. (2007). Adrenergic pharmacology and cognition: Focus on the prefrontal cortex. Pharmacol. Ther..

[77] Zhang H.T., Whisler L.R., Huang Y., Xiang Y., O’Donnell J.M. (2009). Postsynaptic alpha-2 adrenergic receptors are critical for the antidepressant-like effects of desipramine on behavior. Neuropsychopharmacology.

[78] Hein L. (2006). Adrenoceptors and signal transduction in neurons. Cell Tissue Res..

[79] Yamamoto K., Hornykiewicz O. (2004). Proposal for a noradrenaline hypothesis of schizophrenia. Prog. Neuro-Psychopharmacol. Biol. Psychiatry.

[80] Arnsten A.F. (2004). Adrenergic targets for the treatment of cognitive deficits in schizophrenia. Psychopharmacology.

[81] Atzori M., Cuevas-Olguin R., Esquivel-Rendon E., Garcia-Oscos F., Salgado-Delgado R.C., Saderi N., Miranda-Morales M., Treviño M., Pineda J.C., Salgado H. (2016). Locus Ceruleus Norepinephrine Release: A Central Regulator of CNS Spatio-Temporal Activation?. Front. Synaptic. Neurosci..

[82] Birnbaum S.G., Yuan P.X., Wang M., Vijayraghavan S., Bloom A.K., Davis D.J., Gobeske K.T., Sweatt J.D., Manji H.K., Arnsten A.F. (2004). Protein kinase C overactivity impairs prefrontal cortical regulation of working memory. Science.

[83] Oranje B., Glenthøj B.Y. (2013). Clonidine normalizes sensorimotor gating deficits in patients with schizophrenia on stable medication. Schizophr. Bull..

[84] Oranje B., Glenthøj B.Y. (2014). Clonidine normalizes levels of P50 gating in patients with schizophrenia on stable medication. Schizophr. Bull..

[85] Baisley S.K., Fallace K.L., Rajbhandari A.K., Bakshi V.P. (2012). Mutual independence of 5-HT(2) and α1 noradrenergic receptors in mediating deficits in sensorimotor gating. Psychopharmacology.

[86] Friedman J.I., Adler D.N., Temporini H.D., Kemether E., Harvey P.D., White L., Parrella M., Davis K.L. (2001). Guanfacine treatment of cognitive impairment in schizophrenia. Neuropsychopharmacology.

[87] Arnsten A.F. (2009). The Emerging Neurobiology of Attention Deficit Hyperactivity Disorder: The Key Role of the Prefrontal Association Cortex. J. Pediatr..

[88] Gamo N.J., Lur G., Higley M.J., Wang M., Paspalas C.D., Vijayraghavan S., Yang Y., Ramos B.P., Peng K., Kata A. (2015). Stress Impairs Prefrontal Cortical Function via D1 Dopamine Receptor Interactions With Hyperpolarization-Activated Cyclic Nucleotide-Gated Channels. Biol. Psychiatry.

[89] Phillips W.A., Larkum M.E., Harley C.W., Silverstein S.M. (2016). The effects of arousal on apical amplification and conscious state. Neurosci. Conscious..

[90] Wang M., Gamo N.J., Yang Y., Jin L.E., Wang X.J., Laubach M., Mazer J.A., Lee D., Arnsten A.F. (2011). Neuronal basis of age-related working memory decline. Nature.

[91] Valero-Aracama M.J., Reboreda A., Arboit A., Sauvage M., Yoshida M. (2021). Noradrenergic suppression of persistent firing in hippocampal CA1 pyramidal cells through cAMP-PKA pathway. eNeuro.

[92] Wang M., Ramos B.P., Paspalas C.D., Shu Y., Simen A., Duque A., Vijayraghavan S., Brennan A., Dudley A., Nou E. (2007). Alpha2A-adrenoceptors strengthen working memory networks by inhibiting cAMP-HCN channel signaling in prefrontal cortex. Cell.

[93] Arnsten A.F., Jin L.E. (2012). Guanfacine for the treatment of cognitive disorders: A century of discoveries at Yale. Yale J. Biol. Med..

[94] O’Dell T.J., Connor S.A., Guglietta R., Nguyen P.V. (2015). β-Adrenergic receptor signaling and modulation of long-term potentiation in the mammalian hippocampus. Learn. Mem..

[95] Zhou H.C., Sun Y.Y., Cai W., He X.T., Yi F., Li B.M., Zhang X.H. (2013). Activation of β2-adrenoceptor enhances synaptic potentiation and behavioral memory via cAMP-PKA signaling in the medial prefrontal cortex of rats. Learn. Mem..

[96] Shek E., Bardhan S., Cheine M.V., Ahonen J., Wahlbeck K. (2010). Beta-blocker supplementation of standard drug treatment for schizophrenia. Schizophr. Bull..

[97] Mico’ U., Bruno A., Pandolfo G., Maria Romeo V., Mallamace D., D’Arrigo C., Spina E., Zoccali R.A., Muscatello M.R. (2011). Duloxetine as adjunctive treatment to clozapine in patients with schizophrenia: A randomized, placebo-controlled trial. Int. Clin. Psychopharmacol..

[98] Terevnikov V., Stenberg J.H., Joffe M., Tiihonen J., Burkin M., Tchoukhine E., Joffe G. (2010). More evidence on additive antipsychotic effect of adjunctive mirtazapine in schizophrenia: An extension phase of a randomized controlled trial. Hum. Psychopharmacol..

[99] Abbasi S.H., Behpournia H., Ghoreshi A., Salehi B., Raznahan M., Rezazadeh S.A., Rezaei F., Akhondzadeh S. (2010). The effect of mirtazapine add on therapy to risperidone in the treatment of schizophrenia: A double-blind randomized placebo-controlled trial. Schizophr. Res..

[100] Boyda H.N., Ho A.A., Tse L., Procyshyn R.M., Yuen J.W.Y., Kim D.D., Honer W.G., Barr A.M. (2020). Differential Effects of Acute Treatment With Antipsychotic Drugs on Peripheral Catecholamines. Front. Psychiatry.

[101] Clark D.A., Arranz M.J., Mata I., Lopéz-Ilundain J., Pérez-Nievas F., Kerwin R.W. (2005). Polymorphisms in the promoter region of the alpha1A-adrenoceptor gene are associated with schizophrenia/schizoaffective disorder in a Spanish isolate population. Biol. Psychiatry.

[102] Lochman J., Plesník J., Janout V., Povová J., Míšek I., Dvořáková D., Šerý O. (2013). Interactive effect of MTHFR and ADRA2A gene polymorphisms on pathogenesis of schizophrenia. Neuro Endocrinol. Lett..

[103] Wang L.J., Lee S.Y., Chen S.L., Chang Y.H., Chen P.S., Huang S.Y., Tzeng N.S., Chen K.C., Lee I.H., Wang T.Y. (2015). A potential interaction between COMT and MTHFR genetic variants in Han Chinese patients with bipolar II disorder. Sci. Rep..

[104] Picciotto M.R., Higley M.J., Mineur Y.S. (2012). Acetylcholine as a neuromodulator: Cholinergic signaling shapes nervous system function and behavior. Neuron.

[105] Eglen R.M. (2005). Muscarinic receptor subtype pharmacology and physiology. Prog. Med. Chem..

[106] Resende R.R., Adhikari A. (2009). Cholinergic receptor pathways involved in apoptosis, cell proliferation and neuronal differentiation. Cell Commun. Signal..

[107] Espada S., Rojo A.I., Salinas M., Cuadrado A. (2009). The muscarinic M1 receptor activates Nrf2 through a signaling cascade that involves protein kinase C and inhibition of GSK-3beta: Connecting neurotransmission with neuroprotection. J. Neurochem..

[108] Rouse S.T., Hamilton S.E., Potter L.T., Nathanson N.M., Conn P.J. (2000). Muscarinic-induced modulation of potassium conductances is unchanged in mouse hippocampal pyramidal cells that lack functional M1 receptors. Neurosci. Lett..

[109] Volpicelli L.A., Levey A.I. (2004). Muscarinic acetylcholine receptor subtypes in cerebral cortex and hippocampus. Prog. Brain Res..

[110] Hersch S.M., Levey A.I. (1995). Diverse pre- and post-synaptic expression of m1-m4 muscarinic receptor proteins in neurons and afferents in the rat neostriatum. Life Sci..

[111] Nair A.G., Castro L.R.V., El Khoury M., Gorgievski V., Giros B., Tzavara E.T., Hellgren-Kotaleski J., Vincent P. (2019). The high efficacy of muscarinic M4 receptor in D1 medium spiny neurons reverses striatal hyperdopaminergia. Neuropharmacology.

[112] Erskine D., Taylor J.P., Bakker G., Brown A.J.H., Tasker T., Nathan P.J. (2019). Cholinergic muscarinic M. Drug Discov. Today.

[113] Scarr E., Sundram S., Keriakous D., Dean B. (2007). Altered hippocampal muscarinic M4, but not M1, receptor expression from subjects with schizophrenia. Biol. Psychiatry.

[114] Gibbons A.S., Scarr E., Boer S., Money T., Jeon W.J., Felder C., Dean B. (2013). Widespread decreases in cortical muscarinic receptors in a subset of people with schizophrenia. Int. J. Neuropsychopharmacol..

[115] Bakker G., Vingerhoets C., Boucherie D., Caan M., Bloemen O., Eersels J., Booij J., van Amelsvoort T. (2018). Relationship between muscarinic M. Neuroimage Clin..

[116] Odagaki Y., Kinoshita M., Meana J.J., Callado L.F., García-Sevilla J.A. (2020). Functional coupling of M. Eur. Arch. Psychiatry Clin. Neurosci..

[117] Newell K.A., Zavitsanou K., Jew S.K., Huang X.F. (2007). Alterations of muscarinic and GABA receptor binding in the posterior cingulate cortex in schizophrenia. Prog. Neuropsychopharmacol. Biol. Psychiatry.

[118] Zavitsanou K., Katerina Z., Katsifis A., Andrew K., Mattner F., Filomena M., Huang X.F., Xu-Feng H. (2004). Investigation of m1/m4 muscarinic receptors in the anterior cingulate cortex in schizophrenia, bipolar disorder, and major depression disorder. Neuropsychopharmacology.

[119] Ghoshal A., Rook J.M., Dickerson J.W., Roop G.N., Morrison R.D., Jalan-Sakrikar N., Lamsal A., Noetzel M.J., Poslusney M.S., Wood M.R. (2016). Potentiation of M1 Muscarinic Receptor Reverses Plasticity Deficits and Negative and Cognitive Symptoms in a Schizophrenia Mouse Model. Neuropsychopharmacology.

[120] Scarr E., Keriakous D., Crossland N., Dean B. (2006). No change in cortical muscarinic M2, M3 receptors or [35S]GTPgammaS binding in schizophrenia. Life Sci..

[121] Abad N.H., Doulatabad N.S., Mohammadi A., Srazi H.R. (2011). Treatment of Visual Hallucinations in Schizophrenia by Acetylcholinesterase Inhibitors: A case report. Iran. J. Psychiatry.

[122] Patel S.S., Attard A., Jacobsen P., Shergill S. (2010). Acetylcholinesterase Inhibitors (AChEI’s) for the treatment of visual hallucinations in schizophrenia: A case report. BMC Psychiatry.

[123] Buchanan R.W., Conley R.R., Dickinson D., Ball M.P., Feldman S., Gold J.M., McMahon R.P. (2008). Galantamine for the treatment of cognitive impairments in people with schizophrenia. Am. J. Psychiatry.

[124] Dyer M.A., Freudenreich O., Culhane M.A., Pachas G.N., Deckersbach T., Murphy E., Goff D.C., Evins A.E. (2008). High-dose galantamine augmentation inferior to placebo on attention, inhibitory control and working memory performance in nonsmokers with schizophrenia. Schizophr. Res..

[125] Keefe R.S., Malhotra A.K., Meltzer H.Y., Kane J.M., Buchanan R.W., Murthy A., Sovel M., Li C., Goldman R. (2008). Efficacy and safety of donepezil in patients with schizophrenia or schizoaffective disorder: Significant placebo/practice effects in a 12-week, randomized, double-blind, placebo-controlled trial. Neuropsychopharmacology.

[126] Scarr E., Gibbons A.S., Neo J., Udawela M., Dean B. (2013). Cholinergic connectivity: It’s implications for psychiatric disorders. Front. Cell. Neurosci..

[127] Ellis J.R., Ellis K.A., Bartholomeusz C.F., Harrison B.J., Wesnes K.A., Erskine F.F., Vitetta L., Nathan P.J. (2006). Muscarinic and nicotinic receptors synergistically modulate working memory and attention in humans. Int. J. Neuropsychopharmacol..

[128] Klinkenberg I., Blokland A. (2010). The validity of scopolamine as a pharmacological model for cognitive impairment: A review of animal behavioral studies. Neurosci. Biobehav. Rev..

[129] Sambeth A., Riedel W.J., Klinkenberg I., Kähkönen S., Blokland A. (2015). Biperiden selectively induces memory impairment in healthy volunteers: No interaction with citalopram. Psychopharmacology.

[130] Bradley S.R., Lameh J., Ohrmund L., Son T., Bajpai A., Nguyen D., Friberg M., Burstein E.S., Spalding T.A., Ott T.R. (2010). AC-260584, an orally bioavailable M(1) muscarinic receptor allosteric agonist, improves cognitive performance in an animal model. Neuropharmacology.

[131] Fernández de Sevilla D., Núñez A., Borde M., Malinow R., Buño W. (2008). Cholinergic-mediated IP3-receptor activation induces long-lasting synaptic enhancement in CA1 pyramidal neurons. J. Neurosci..

[132] Brown D.A. (2018). Regulation of neural ion channels by muscarinic receptors. Neuropharmacology.

[133] Giessel A.J., Sabatini B.L. (2010). M1 muscarinic receptors boost synaptic potentials and calcium influx in dendritic spines by inhibiting postsynaptic SK channels. Neuron.

[134] Buchanan K.A., Petrovic M.M., Chamberlain S.E., Marrion N.V., Mellor J.R. (2010). Facilitation of long-term potentiation by muscarinic M(1) receptors is mediated by inhibition of SK channels. Neuron.

[135] Zhao L.X., Ge Y.H., Li J.B., Xiong C.H., Law P.Y., Xu J.R., Qiu Y., Chen H.Z. (2019). M1 muscarinic receptors regulate the phosphorylation of AMPA receptor subunit GluA1. FASEB J..

[136] Zhao L.X., Ge Y.H., Xiong C.H., Tang L., Yan Y.H., Law P.Y., Qiu Y., Chen H.Z. (2018). M1 muscarinic receptor facilitates cognitive function by interplay with AMPA receptor GluA1 subunit. FASEB J..

[137] Zeppillo T., Schulmann A., Macciardi F., Hjelm B.E., Föcking M., Sequeira P.A., Guella I., Cotter D., Bunney W.E., Limon A. (2020). Functional impairment of cortical AMPA receptors in schizophrenia. Schizophr Res..

[138] Gururajan A., van den Buuse M. (2014). Is the mTOR-signalling cascade disrupted in Schizophrenia?. J. Neurochem..

[139] Jeon J., Dencker D., Wörtwein G., Woldbye D.P., Cui Y., Davis A.A., Levey A.I., Schütz G., Sager T.N., Mørk A. (2010). A subpopulation of neuronal M4 muscarinic acetylcholine receptors plays a critical role in modulating dopamine-dependent behaviors. J. Neurosci..

[140] Berger M., Gray J.A., Roth B.L. (2009). The expanded biology of serotonin. Annu. Rev. Med..

[141] Palacios J.M. (2016). Serotonin receptors in brain revisited. Brain Res..

[142] Hoyer D., Hannon J.P., Martin G.R. (2002). Molecular, pharmacological and functional diversity of 5-HT receptors. Pharmacol. Biochem. Behav..

[143] Geyer M.A., Vollenweider F.X. (2008). Serotonin research: Contributions to understanding psychoses. Trends Pharmacol. Sci..

[144] Green A.R. (2006). Neuropharmacology of 5-hydroxytryptamine. Br. J. Pharmacol..

[145] González-Maeso J., Weisstaub N.V., Zhou M., Chan P., Ivic L., Ang R., Lira A., Bradley-Moore M., Ge Y., Zhou Q. (2007). Hallucinogens recruit specific cortical 5-HT(2A) receptor-mediated signaling pathways to affect behavior. Neuron.

[146] González-Maeso J., Yuen T., Ebersole B.J., Wurmbach E., Lira A., Zhou M., Weisstaub N., Hen R., Gingrich J.A., Sealfon S.C. (2003). Transcriptome fingerprints distinguish hallucinogenic and nonhallucinogenic 5-hydroxytryptamine 2A receptor agonist effects in mouse somatosensory cortex. J. Neurosci..

[147] Mahesh G., Jaiswal P., Dey S., Sengupta J., Mukherjee S. (2018). Cloning, Expression, Purification and Characterization of Oligomeric States of the Native 5HT2A G-Protein-Coupled Receptor. Protein Pept. Lett..

[148] Rasmussen H., Frokjaer V.G., Hilker R.W., Madsen J., Anhøj S., Oranje B., Pinborg L.H., Glenthøj B., Knudsen G.M. (2016). Low frontal serotonin 2A receptor binding is a state marker for schizophrenia?. Eur. Neuropsychopharmacol..

[149] Liégeois J.F., Ichikawa J., Meltzer H.Y. (2002). 5-HT(2A) receptor antagonism potentiates haloperidol-induced dopamine release in rat medial prefrontal cortex and inhibits that in the nucleus accumbens in a dose-dependent manner. Brain Res..

[150] Meltzer H.Y. (2004). What’s atypical about atypical antipsychotic drugs?. Curr. Opin. Pharmacol..

[151] Kapur S., Remington G. (2001). Dopamine D(2) receptors and their role in atypical antipsychotic action: Still necessary and may even be sufficient. Biol. Psychiatry.

[152] Kapur S., Wadenberg M.L., Remington G. (2000). Are animal studies of antipsychotics appropriately dosed? Lessons from the bedside to the bench. Can. J. Psychiatry.

[153] Zhang C., Li Q., Meng L., Ren Y. (2020). Design of novel dopamine D. J. Biomol. Struct. Dyn..

[154] Krause M., Zhu Y., Huhn M., Schneider-Thoma J., Bighelli I., Nikolakopoulou A., Leucht S. (2018). Antipsychotic drugs for patients with schizophrenia and predominant or prominent negative symptoms: A systematic review and meta-analysis. Eur. Arch. Psychiatry Clin. Neurosci..

[155] Tarsy D., Baldessarini R.J., Tarazi F.I. (2002). Effects of newer antipsychotics on extrapyramidal function. CNS Drugs.

[156] Meltzer H.Y., Li Z., Kaneda Y., Ichikawa J. (2003). Serotonin receptors: Their key role in drugs to treat schizophrenia. Prog Neuropsychopharmacol. Biol. Psychiatry.

[157] McOmish C.E., Lira A., Hanks J.B., Gingrich J.A. (2012). Clozapine-induced locomotor suppression is mediated by 5-HT2A receptors in the forebrain. Neuropsychopharmacology.

[158] Creed-Carson M., Oraha A., Nobrega J.N. (2011). Effects of 5-HT(2A) and 5-HT(2C) receptor antagonists on acute and chronic dyskinetic effects induced by haloperidol in rats. Behav. Brain Res..

[159] Tsartsalis S., Tournier B.B., Gloria Y., Millet P., Ginovart N. (2021). Effect of 5-HT2A receptor antagonism on levels of D2/3 receptor occupancy and adverse behavioral side-effects induced by haloperidol: A SPECT imaging study in the rat. Transl. Psychiatry.

[160] González-Maeso J., Ang R.L., Yuen T., Chan P., Weisstaub N.V., López-Giménez J.F., Zhou M., Okawa Y., Callado L.F., Milligan G. (2008). Identification of a serotonin/glutamate receptor complex implicated in psychosis. Nature.

[161] Moreno J.L., Miranda-Azpiazu P., García-Bea A., Younkin J., Cui M., Kozlenkov A., Ben-Ezra A., Voloudakis G., Fakira A.K., Baki L. (2016). Allosteric signaling through an mGlu2 and 5-HT2A heteromeric receptor complex and its potential contribution to schizophrenia. Sci. Signal..

[162] Shah U.H., González-Maeso J. (2019). Serotonin and Glutamate Interactions in Preclinical Schizophrenia Models. ACS Chem. Neurosci..

[163] Moreno J.L., Holloway T., Albizu L., Sealfon S.C., González-Maeso J. (2011). Metabotropic glutamate mGlu2 receptor is necessary for the pharmacological and behavioral effects induced by hallucinogenic 5-HT2A receptor agonists. Neurosci. Lett..

[164] Selvaraj S., Arnone D., Cappai A., Howes O. (2014). Alterations in the serotonin system in schizophrenia: A systematic review and meta-analysis of postmortem and molecular imaging studies. Neurosci. Biobehav. Rev..

[165] Yasuno F., Suhara T., Ichimiya T., Takano A., Ando T., Okubo Y. (2004). Decreased 5-HT1A receptor binding in amygdala of schizophrenia. Biol. Psychiatry.

[166] Díaz-Mataix L., Scorza M.C., Bortolozzi A., Toth M., Celada P., Artigas F. (2005). Involvement of 5-HT1A receptors in prefrontal cortex in the modulation of dopaminergic activity: Role in atypical antipsychotic action. J. Neurosci..

[167] Schotte A., Janssen P.F., Gommeren W., Luyten W.H., Van Gompel P., Lesage A.S., De Loore K., Leysen J.E. (1996). Risperidone compared with new and reference antipsychotic drugs: In vitro and in vivo receptor binding. Psychopharmacology.

[168] Reavill C., Rogers D.C. (2001). The therapeutic potential of 5-HT6 receptor antagonists. Curr. Opin. Investig. Drugs.

[169] Nikiforuk A. (2016). Serotonergic and Cholinergic Strategies as Potential Targets for the Treatment of Schizophrenia. Curr. Pharm. Des..

[170] Murray R.M., Bhavsar V., Tripoli G., Howes O. (2017). 30 Years on: How the Neurodevelopmental Hypothesis of Schizophrenia Morphed Into the Developmental Risk Factor Model of Psychosis. Schizophr. Bull..

[171] Owen M.J., O’Donovan M.C., Thapar A., Craddock N. (2011). Neurodevelopmental hypothesis of schizophrenia. Br. J. Psychiatry.

[172] Reynolds G.P., Yao Z., Zhang X., Sun J., Zhang Z. (2005). Pharmacogenetics of treatment in first-episode schizophrenia: D3 and 5-HT2C receptor polymorphisms separately associate with positive and negative symptom response. Eur. Neuropsychopharmacol..

[173] Arranz M., Collier D., Sodhi M., Ball D., Roberts G., Price J., Sham P., Kerwin R. (1995). Association between clozapine response and allelic variation in 5-HT2A receptor gene. Lancet.

[174] Kim J.H., Marton J., Ametamey S.M., Cumming P. (2020). A Review of Molecular Imaging of Glutamate Receptors. Molecules.

[175] Reiner A., Levitz J. (2018). Glutamatergic Signaling in the Central Nervous System: Ionotropic and Metabotropic Receptors in Concert. Neuron.

[176] Crupi R., Impellizzeri D., Cuzzocrea S. (2019). Role of Metabotropic Glutamate Receptors in Neurological Disorders. Front. Mol. Neurosci..

[177] Hermans E., Challiss R.A. (2001). Structural, signalling and regulatory properties of the group I metabotropic glutamate receptors: Prototypic family C G-protein-coupled receptors. Biochem. J..

[178] Page G., Khidir F.A., Pain S., Barrier L., Fauconneau B., Guillard O., Piriou A., Hugon J. (2006). Group I metabotropic glutamate receptors activate the p70S6 kinase via both mammalian target of rapamycin (mTOR) and extracellular signal-regulated kinase (ERK 1/2) signaling pathways in rat striatal and hippocampal synaptoneurosomes. Neurochem. Int..

[179] Correa A.M.B., Guimarães J.D.S., Dos Santos E Alhadas E., Kushmerick C. (2017). Control of neuronal excitability by Group I metabotropic glutamate receptors. Biophys. Rev..

[180] Ayoub M.A., Angelicheva D., Vile D., Chandler D., Morar B., Cavanaugh J.A., Visscher P.M., Jablensky A., Pfleger K.D., Kalaydjieva L. (2012). Deleterious GRM1 mutations in schizophrenia. PLoS ONE.

[181] Volk D.W., Eggan S.M., Lewis D.A. (2010). Alterations in metabotropic glutamate receptor 1α and regulator of G protein signaling 4 in the prefrontal cortex in schizophrenia. Am. J. Psychiatry.

[182] Niswender C.M., Conn P.J. (2010). Metabotropic glutamate receptors: Physiology, pharmacology, and disease. Annu. Rev. Pharmacol. Toxicol..

[183] Maksymetz J., Moran S.P., Conn P.J. (2017). Targeting metabotropic glutamate receptors for novel treatments of schizophrenia. Mol. Brain.

[184] Balu D.T., Li Y., Takagi S., Presti K.T., Ramikie T.S., Rook J.M., Jones C.K., Lindsley C.W., Conn P.J., Bolshakov V.Y. (2016). An mGlu5-Positive Allosteric Modulator Rescues the Neuroplasticity Deficits in a Genetic Model of NMDA Receptor Hypofunction in Schizophrenia. Neuropsychopharmacology.

[185] Ohishi H., Shigemoto R., Nakanishi S., Mizuno N. (1993). Distribution of the mRNA for a metabotropic glutamate receptor (mGluR3) in the rat brain: An in situ hybridization study. J. Comp. Neurol..

[186] Mazzitelli M., Palazzo E., Maione S., Neugebauer V. (2018). Group II Metabotropic Glutamate Receptors: Role in Pain Mechanisms and Pain Modulation. Front. Mol. Neurosci.

[187] Uslaner J.M., Smith S.M., Huszar S.L., Pachmerhiwala R., Hinchliffe R.M., Vardigan J.D., Hutson P.H. (2009). Combined administration of an mGlu2/3 receptor agonist and a 5-HT 2A receptor antagonist markedly attenuate the psychomotor-activating and neurochemical effects of psychostimulants. Psychopharmacology.

[188] Moghaddam B., Adams B.W. (1998). Reversal of phencyclidine effects by a group II metabotropic glutamate receptor agonist in rats. Science.

[189] Cartmell J., Monn J.A., Schoepp D.D. (2000). Attenuation of specific PCP-evoked behaviors by the potent mGlu2/3 receptor agonist, LY379268 and comparison with the atypical antipsychotic, clozapine. Psychopharmacology.

[190] Krystal J.H., Abi-Saab W., Perry E., D’Souza D.C., Liu N., Gueorguieva R., McDougall L., Hunsberger T., Belger A., Levine L. (2005). Preliminary evidence of attenuation of the disruptive effects of the NMDA glutamate receptor antagonist, ketamine, on working memory by pretreatment with the group II metabotropic glutamate receptor agonist, LY354740, in healthy human subjects. Psychopharmacology.

[191] Patil S.T., Zhang L., Martenyi F., Lowe S.L., Jackson K.A., Andreev B.V., Avedisova A.S., Bardenstein L.M., Gurovich I.Y., Morozova M.A. (2007). Activation of mGlu2/3 receptors as a new approach to treat schizophrenia: A randomized Phase 2 clinical trial. Nat. Med..

[192] Moreno J.L., Sealfon S.C., González-Maeso J. (2009). Group II metabotropic glutamate receptors and schizophrenia. Cell. Mol. Life Sci..

[193] Galici R., Echemendia N.G., Rodriguez A.L., Conn P.J. (2005). A selective allosteric potentiator of metabotropic glutamate (mGlu) 2 receptors has effects similar to an orthosteric mGlu2/3 receptor agonist in mouse models predictive of antipsychotic activity. J. Pharmacol. Exp. Ther..

[194] Benneyworth M.A., Xiang Z., Smith R.L., Garcia E.E., Conn P.J., Sanders-Bush E. (2007). A selective positive allosteric modulator of metabotropic glutamate receptor subtype 2 blocks a hallucinogenic drug model of psychosis. Mol. Pharmacol..

[195] Kinon B.J., Gómez J.C. (2013). Clinical development of pomaglumetad methionil: A non-dopaminergic treatment for schizophrenia. Neuropharmacology.

[196] Adams D.H., Kinon B.J., Baygani S., Millen B.A., Velona I., Kollack-Walker S., Walling D.P. (2013). A long-term, phase 2, multicenter, randomized, open-label, comparative safety study of pomaglumetad methionil (LY2140023 monohydrate) versus atypical antipsychotic standard of care in patients with schizophrenia. BMC Psychiatry.

[197] Hopkins C.R. (2013). Is there a path forward for mGlu(2) positive allosteric modulators for the treatment of schizophrenia?. ACS Chem. Neurosci..

[198] Salih H., Anghelescu I., Kezic I., Sinha V., Hoeben E., Van Nueten L., De Smedt H., De Boer P. (2015). Pharmacokinetic and pharmacodynamic characterisation of JNJ-40411813, a positive allosteric modulator of mGluR2, in two randomised, double-blind phase-I studies. J. Psychopharmacol..

[199] Litman R.E., Smith M.A., Doherty J.J., Cross A., Raines S., Gertsik L., Zukin S.R. (2016). AZD8529, a positive allosteric modulator at the mGluR2 receptor, does not improve symptoms in schizophrenia: A proof of principle study. Schizophr. Res..

[200] Iacovelli L., Bruno V., Salvatore L., Melchiorri D., Gradini R., Caricasole A., Barletta E., De Blasi A., Nicoletti F. (2002). Native group-III metabotropic glutamate receptors are coupled to the mitogen-activated protein kinase/phosphatidylinositol-3-kinase pathways. J. Neurochem..

[201] Senter R.K., Ghoshal A., Walker A.G., Xiang Z., Niswender C.M., Conn P.J. (2016). The Role of mGlu Receptors in Hippocampal Plasticity Deficits in Neurological and Psychiatric Disorders: Implications for Allosteric Modulators as Novel Therapeutic Strategies. Curr. Neuropharmacol..

[202] Mena A., Ruiz-Salas J.C., Puentes A., Dorado I., Ruiz-Veguilla M., De la Casa L.G. (2016). Reduced Prepulse Inhibition as a Biomarker of Schizophrenia. Front. Behav. Neurosci..

[203] Wierońska J.M., Acher F.C., Sławińska A., Gruca P., Lasoń-Tyburkiewicz M., Papp M., Pilc A. (2013). The antipsychotic-like effects of the mGlu group III orthosteric agonist, LSP1-2111, involves 5-HT_1_A signalling. Psychopharmacology.

[204] Woźniak M., Gołembiowska K., Noworyta-Sokołowska K., Acher F., Cieślik P., Kusek M., Tokarski K., Pilc A., Wierońska J.M. (2017). Neurochemical and behavioral studies on the 5-HT. Neuropharmacology.

[205] Sławińska A., Wierońska J.M., Stachowicz K., Marciniak M., Lasoń-Tyburkiewicz M., Gruca P., Papp M., Kusek M., Tokarski K., Doller D. (2013). The antipsychotic-like effects of positive allosteric modulators of metabotropic glutamate mGlu4 receptors in rodents. Br. J. Pharmacol..

[206] Kalinichev M., Le Poul E., Boléa C., Girard F., Campo B., Fonsi M., Royer-Urios I., Browne S.E., Uslaner J.M., Davis M.J. (2014). Characterization of the novel positive allosteric modulator of the metabotropic glutamate receptor 4 ADX88178 in rodent models of neuropsychiatric disorders. J. Pharmacol. Exp. Ther..

[207] O’Connor R.M., Finger B.C., Flor P.J., Cryan J.F. (2010). Metabotropic glutamate receptor 7: At the interface of cognition and emotion. Eur. J. Pharmacol..

[208] Suzuki G., Tsukamoto N., Fushiki H., Kawagishi A., Nakamura M., Kurihara H., Mitsuya M., Ohkubo M., Ohta H. (2007). In vitro pharmacological characterization of novel isoxazolopyridone derivatives as allosteric metabotropic glutamate receptor 7 antagonists. J. Pharmacol. Exp. Ther..

[209] Kalinichev M., Rouillier M., Girard F., Royer-Urios I., Bournique B., Finn T., Charvin D., Campo B., Le Poul E., Mutel V. (2013). ADX71743, a potent and selective negative allosteric modulator of metabotropic glutamate receptor 7: In vitro and in vivo characterization. J. Pharmacol. Exp. Ther..

[210] Cieślik P., Woźniak M., Kaczorowska K., Brański P., Burnat G., Chocyk A., Bobula B., Gruca P., Litwa E., Pałucha-Poniewiera A. (2018). Negative Allosteric Modulators of mGlu. Front. Mol. Neurosci..

[211] Gerlai R., Adams B., Fitch T., Chaney S., Baez M. (2002). Performance deficits of mGluR8 knockout mice in learning tasks: The effects of null mutation and the background genotype. Neuropharmacology.

[212] Duvoisin R.M., Zhang C., Pfankuch T.F., O’Connor H., Gayet-Primo J., Quraishi S., Raber J. (2005). Increased measures of anxiety and weight gain in mice lacking the group III metabotropic glutamate receptor mGluR8. Eur. J. Neurosci..

[213] Davis M.J., Duvoisin R.M., Raber J. (2013). Related functions of mGlu4 and mGlu8. Pharmacol. Biochem. Behav..

[214] Fendt M., Bürki H., Imobersteg S., van der Putten H., McAllister K., Leslie J.C., Shaw D., Hölscher C. (2010). The effect of mGlu8 deficiency in animal models of psychiatric diseases. Genes Brain Behav..

[215] Ossowska K., Pietraszek M., Wardas J., Wolfarth S. (2004). Potential antipsychotic and extrapyramidal effects of (R,S)-3,4-dicarboxyphenylglycine [(R,S)-3,4-DCPG], a mixed AMPA antagonist/mGluR8 agonist. Pol. J. Pharmacol..

[216] Robbins M.J., Starr K.R., Honey A., Soffin E.M., Rourke C., Jones G.A., Kelly F.M., Strum J., Melarange R.A., Harris A.J. (2007). Evaluation of the mGlu8 receptor as a putative therapeutic target in schizophrenia. Brain Res..

[217] Ishikawa M., Mizukami K., Iwakiri M., Asada T. (2005). Immunohistochemical and immunoblot analysis of gamma-aminobutyric acid B receptor in the prefrontal cortex of subjects with schizophrenia and bipolar disorder. Neurosci. Lett..

[218] Fatemi S.H., Folsom T.D., Thuras P.D. (2011). Deficits in GABA(B) receptor system in schizophrenia and mood disorders: A postmortem study. Schizophr. Res..

[219] Chalifoux J.R., Carter A.G. (2010). GABAB receptors modulate NMDA receptor calcium signals in dendritic spines. Neuron.

[220] Fatemi S.H., Folsom T.D., Thuras P.D. (2017). GABA_A_ and GABA_B_ receptor dysregulation in superior frontal cortex of subjects with schizophrenia and bipolar disorder. Synapse.

[221] Li P., Stewart R., Butler A., Gonzalez-Cota A.L., Harmon S., Salkoff L. (2017). GABA-B Controls Persistent Na. eNeuro.

[222] Mizukami K., Ishikawa M., Hidaka S., Iwakiri M., Sasaki M., Iritani S. (2002). Immunohistochemical localization of GABAB receptor in the entorhinal cortex and inferior temporal cortex of schizophrenic brain. Prog. Neuropsychopharmacol. Biol. Psychiatry.

[223] Zai G., King N., Wong G.W., Barr C.L., Kennedy J.L. (2005). Possible association between the gamma-aminobutyric acid type B receptor 1 (GABBR1) gene and schizophrenia. Eur. Neuropsychopharmacol..

[224] Imai K., Harada S., Kawanishi Y., Tachikawa H., Okubo T., Asada T. (2002). Association analysis of an (AC)n repeat polymorphism in the GABA(B) receptor gene and schizophrenia. Am. J. Med. Genet..

[225] Zhao X., Qin S., Shi Y., Zhang A., Zhang J., Bian L., Wan C., Feng G., Gu N., Zhang G. (2007). Systematic study of association of four GABAergic genes: Glutamic acid decarboxylase 1 gene, glutamic acid decarboxylase 2 gene, GABA(B) receptor 1 gene and GABA(A) receptor subunit beta2 gene, with schizophrenia using a universal DNA microarray. Schizophr. Res..

[226] Klempan T.A., Sequeira A., Canetti L., Lalovic A., Ernst C., ffrench-Mullen J., Turecki G. (2009). Altered expression of genes involved in ATP biosynthesis and GABAergic neurotransmission in the ventral prefrontal cortex of suicides with and without major depression. Mol. Psychiatry.

[227] Kantrowitz J., Citrome L., Javitt D. (2009). GABA(B) receptors, schizophrenia and sleep dysfunction: A review of the relationship and its potential clinical and therapeutic implications. CNS Drugs.

[228] Arai S., Takuma K., Mizoguchi H., Ibi D., Nagai T., Takahashi K., Kamei H., Nabeshima T., Yamada K. (2008). Involvement of pallidotegmental neurons in methamphetamine- and MK-801-induced impairment of prepulse inhibition of the acoustic startle reflex in mice: Reversal by GABAB receptor agonist baclofen. Neuropsychopharmacology.

[229] Bortolato M., Frau R., Aru G.N., Orrù M., Gessa G.L. (2004). Baclofen reverses the reduction in prepulse inhibition of the acoustic startle response induced by dizocilpine, but not by apomorphine. Psychopharmacology.

[230] Fejgin K., Pålsson E., Wass C., Finnerty N., Lowry J., Klamer D. (2009). Prefrontal GABA(B) receptor activation attenuates phencyclidine-induced impairments of prepulse inhibition: Involvement of nitric oxide. Neuropsychopharmacology.

[231] Kaupmann K., Cryan J.F., Wellendorph P., Mombereau C., Sansig G., Klebs K., Schmutz M., Froestl W., van der Putten H., Mosbacher J. (2003). Specific gamma-hydroxybutyrate-binding sites but loss of pharmacological effects of gamma-hydroxybutyrate in GABA(B)(1)-deficient mice. Eur. J. Neurosci..

[232] Ma J., Stan Leung L. (2017). Effects of GABA-B receptor positive modulator on ketamine-induced psychosis-relevant behaviors and hippocampal electrical activity in freely moving rats. Psychopharmacology.

[233] Helm K.A., Haberman R.P., Dean S.L., Hoyt E.C., Melcher T., Lund P.K., Gallagher M. (2005). GABAB receptor antagonist SGS742 improves spatial memory and reduces protein binding to the cAMP response element (CRE) in the hippocampus. Neuropharmacology.

[234] Ma J., Leung L.S. (2011). GABA(B) receptor blockade in the hippocampus affects sensory and sensorimotor gating in Long-Evans rats. Psychopharmacology.

[235] Selten M.M., Meyer F., Ba W., Vallès A., Maas D.A., Negwer M., Eijsink V.D., van Vugt R.W.M., van Hulten J.A., van Bakel N.H.M. (2016). Increased GABA_B_ receptor signaling in a rat model for schizophrenia. Sci. Rep..

[236] Wierońska J.M., Kusek M., Tokarski K., Wabno J., Froestl W., Pilc A. (2011). The GABA B receptor agonist CGP44532 and the positive modulator GS39783 reverse some behavioural changes related to positive syndromes of psychosis in mice. Br. J. Pharmacol..

[237] Cedillo L.N., Miranda F. (2013). Effects of co-administration of the GABAB receptor agonist baclofen and a positive allosteric modulator of the GABAB receptor, CGP7930, on the development and expression of amphetamine-induced locomotor sensitization in rats. Pharmacol. Rep..

[238] Nair P.C., McKinnon R.A., Miners J.O., Bastiampillai T. (2020). Binding of clozapine to the GABA. Mol. Psychiatry.

[239] Otmakhova N.A., Lisman J.E. (2004). Contribution of Ih and GABAB to synaptically induced afterhyperpolarizations in CA1: A brake on the NMDA response. J. Neurophysiol..

[240] Pérez-Garci E., Gassmann M., Bettler B., Larkum M.E. (2006). The GABAB1b isoform mediates long-lasting inhibition of dendritic Ca2+ spikes in layer 5 somatosensory pyramidal neurons. Neuron.

[241] Kulik A., Vida I., Luján R., Haas C.A., López-Bendito G., Shigemoto R., Frotscher M. (2003). Subcellular localization of metabotropic GABA(B) receptor subunits GABA(B1a/b) and GABA(B2) in the rat hippocampus. J. Neurosci..

[242] Bachelerie F., Ben-Baruch A., Burkhardt A.M., Combadiere C., Farber J.M., Graham G.J., Horuk R., Sparre-Ulrich A.H., Locati M., Luster A.D. (2014). International Union of Basic and Clinical Pharmacology. [corrected]. LXXXIX. Update on the extended family of chemokine receptors and introducing a new nomenclature for atypical chemokine receptors. Pharmacol. Rev..

[243] Eiger D.S., Boldizsar N., Honeycutt C.C., Gardner J., Rajagopal S. (2021). Biased agonism at chemokine receptors. Cell. Signal..

[244] Bachelerie F., Graham G.J., Locati M., Mantovani A., Murphy P.M., Nibbs R., Rot A., Sozzani S., Thelen M. (2014). New nomenclature for atypical chemokine receptors. Nat. Immunol..

[245] Bonecchi R., Graham G.J. (2016). Atypical Chemokine Receptors and Their Roles in the Resolution of the Inflammatory Response. Front. Immunol..

[246] Tian X., Kang D.S., Benovic J.L. (2014). β-arrestins and G protein-coupled receptor trafficking. Handb. Exp. Pharmacol..

[247] Rajagopal S., Rajagopal K., Lefkowitz R.J. (2010). Teaching old receptors new tricks: Biasing seven-transmembrane receptors. Nat. Rev. Drug Discov..

[248] Bennett L.D., Fox J.M., Signoret N. (2011). Mechanisms regulating chemokine receptor activity. Immunology.

[249] Hughes C.E., Nibbs R.J.B. (2018). A guide to chemokines and their receptors. FEBS J..

[250] Stone M.J., Hayward J.A., Huang C., E Huma Z., Sanchez J. (2017). Mechanisms of Regulation of the Chemokine-Receptor Network. Int. J. Mol. Sci..

[251] Stephens B., Handel T.M. (2013). Chemokine receptor oligomerization and allostery. Prog. Mol. Biol. Transl. Sci..

[252] Yang L.K., Hou Z.S., Tao Y.X. (2021). Biased signaling in naturally occurring mutations of G protein-coupled receptors associated with diverse human diseases. Biochim. Biophys. Acta Mol. Basis Dis..

[253] Rostène W., Dansereau M.A., Godefroy D., Van Steenwinckel J., Reaux-Le Goazigo A., Mélik-Parsadaniantz S., Apartis E., Hunot S., Beaudet N., Sarret P. (2011). Neurochemokines: A menage a trois providing new insights on the functions of chemokines in the central nervous system. J. Neurochem..

[254] Rostène W., Guyon A., Kular L., Godefroy D., Barbieri F., Bajetto A., Banisadr G., Callewaere C., Conductier G., Rovère C. (2011). Chemokines and chemokine receptors: New actors in neuroendocrine regulations. Front. Neuroendocrinol..

[255] Sweeney M.D., Zhao Z., Montagne A., Nelson A.R., Zlokovic B.V. (2019). Blood-Brain Barrier: From Physiology to Disease and Back. Physiol. Rev..

[256] Najjar S., Pahlajani S., De Sanctis V., Stern J.N.H., Najjar A., Chong D. (2017). Neurovascular Unit Dysfunction and Blood-Brain Barrier Hyperpermeability Contribute to Schizophrenia Neurobiology: A Theoretical Integration of Clinical and Experimental Evidence. Front. Psychiatry.

[257] Ochoa S., Usall J., Cobo J., Labad X., Kulkarni J. (2012). Gender differences in schizophrenia and first-episode psychosis: A comprehensive literature review. Schizophr. Res. Treat..

[258] Mendrek A., Mancini-Marïe A. (2016). Sex/gender differences in the brain and cognition in schizophrenia. Neurosci. Biobehav. Rev..

[259] Cartier L., Hartley O., Dubois-Dauphin M., Krause K.H. (2005). Chemokine receptors in the central nervous system: Role in brain inflammation and neurodegenerative diseases. Brain Res. Brain Res. Rev..

[260] Pedemonte E., Mancardi G., Giunti D., Corcione A., Benvenuto F., Pistoia V., Uccelli A. (2006). Mechanisms of the adaptive immune response inside the central nervous system during inflammatory and autoimmune diseases. Pharmacol. Ther..

[261] Ivanovska M., Abdi Z., Murdjeva M., Macedo D., Maes A., Maes M. (2020). CCL-11 or Eotaxin-1: An Immune Marker for Ageing and Accelerated Ageing in Neuro-Psychiatric Disorders. Pharmaceuticals.

[262] Sirivichayakul S., Kanchanatawan B., Thika S., Carvalho A.F., Maes M. (2019). Eotaxin, an Endogenous Cognitive Deteriorating Chemokine (ECDC), Is a Major Contributor to Cognitive Decline in Normal People and to Executive, Memory, and Sustained Attention Deficits, Formal Thought Disorders, and Psychopathology in Schizophrenia Patients. Neurotox. Res..

[263] Pedrini M., Massuda R., de Lucena D., Macêdo D., Paz A.V., Lobato M.I., Belmonte-de-Abreu P.S., Ceresér K.M., Rocha N.P., Curra M.D. (2014). Differences in eotaxin serum levels patients with recent onset and in chronic stable schizophrenia: A clue for understanding accelerating aging profile. Schizophr. Res..

[264] Frydecka D., Krzystek-Korpacka M., Lubeiro A., Stramecki F., Stańczykiewicz B., Beszłej J.A., Piotrowski P., Kotowicz K., Szewczuk-Bogusławska M., Pawlak-Adamska E. (2018). Profiling inflammatory signatures of schizophrenia: A cross-sectional and meta-analysis study. Brain Behav. Immun..

[265] Al-Hakeim H.K., Almulla A.F., Maes M. (2020). The Neuroimmune and Neurotoxic Fingerprint of Major Neurocognitive Psychosis or Deficit Schizophrenia: A Supervised Machine Learning Study. Neurotox. Res..

[266] Al-Dujaili A.H., Mousa R.F., Al-Hakeim H.K., Maes M. (2021). High Mobility Group Protein 1 and Dickkopf-Related Protein 1 in Schizophrenia and Treatment-Resistant Schizophrenia: Associations With Interleukin-6, Symptom Domains, and Neurocognitive Impairments. Schizophr. Bull..

[267] Teixeira A.L., Reis H.J., Nicolato R., Brito-Melo G., Correa H., Teixeira M.M., Romano-Silva M.A. (2008). Increased serum levels of CCL11/eotaxin in schizophrenia. Prog. Neuropsychopharmacol. Biol. Psychiatry.

[268] Cronshaw D.G., Kouroumalis A., Parry R., Webb A., Brown Z., Ward S.G. (2006). Evidence that phospholipase-C-dependent, calcium-independent mechanisms are required for directional migration of T-lymphocytes in response to the CCR4 ligands CCL17 and CCL22. J. Leukoc. Biol..

[269] Smit M.J., Verdijk P., van der Raaij-Helmer E.M., Navis M., Hensbergen P.J., Leurs R., Tensen C.P. (2003). CXCR3-mediated chemotaxis of human T cells is regulated by a Gi- and phospholipase C-dependent pathway and not via activation of MEK/p44/p42 MAPK nor Akt/PI-3 kinase. Blood.

[270] Soriano S.F., Serrano A., Hernanz-Falcón P., Martín de Ana A., Monterrubio M., Martínez C., Rodríguez-Frade J.M., Mellado M. (2003). Chemokines integrate JAK/STAT and G-protein pathways during chemotaxis and calcium flux responses. Eur. J. Immunol..

[271] Fillman S.G., Cloonan N., Miller L.C., Weickert C.S. (2013). Markers of inflammation in the prefrontal cortex of individuals with schizophrenia. Mol. Psychiatry.

[272] Brown A.S. (2006). Prenatal infection as a risk factor for schizophrenia. Schizophr. Bull..

[273] Ellman L.M., Deicken R.F., Vinogradov S., Kremen W.S., Poole J.H., Kern D.M., Tsai W.Y., Schaefer C.A., Brown A.S. (2010). Structural brain alterations in schizophrenia following fetal exposure to the inflammatory cytokine interleukin-8. Schizophr. Res..

[274] Martinelli R., Sabroe I., LaRosa G., Williams T.J., Pease J.E. (2001). The CC chemokine eotaxin (CCL11) is a partial agonist of CC chemokine receptor 2b. J. Biol. Chem..

[275] Borroto-Escuela D.O., Tarakanov A.O., Bechter K., Fuxe K. (2017). IL1R2, CCR2, and CXCR4 May Form Heteroreceptor Complexes with NMDAR and D2R: Relevance for Schizophrenia. Front. Psychiatry.

[276] Bazan J.F., Bacon K.B., Hardiman G., Wang W., Soo K., Rossi D., Greaves D.R., Zlotnik A., Schall T.J. (1997). A new class of membrane-bound chemokine with a CX3C motif. Nature.

[277] Cardona A.E., Sasse M.E., Liu L., Cardona S.M., Mizutani M., Savarin C., Hu T., Ransohoff R.M. (2008). Scavenging roles of chemokine receptors: Chemokine receptor deficiency is associated with increased levels of ligand in circulation and tissues. Blood.

[278] Reshef R., Kudryavitskaya E., Shani-Narkiss H., Isaacson B., Rimmerman N., Mizrahi A., Yirmiya R. (2017). The role of microglia and their CX3CR1 signaling in adult neurogenesis in the olfactory bulb. Elife.

[279] Chamera K., Trojan E., Szuster-Głuszczak M., Basta-Kaim A. (2020). The Potential Role of Dysfunctions in Neuron-Microglia Communication in the Pathogenesis of Brain Disorders. Curr. Neuropharmacol..

[280] Pawelec P., Ziemka-Nalecz M., Sypecka J., Zalewska T. (2020). The Impact of the CX3CL1/CX3CR1 Axis in Neurological Disorders. Cells.

[281] Stuart M.J., Singhal G., Baune B.T. (2015). Systematic Review of the Neurobiological Relevance of Chemokines to Psychiatric Disorders. Front. Cell. Neurosci..

[282] Asevedo E., Gadelha A., Noto C., Mansur R.B., Zugman A., Belangero S.I., Berberian A.A., Scarpato B.S., Leclerc E., Teixeira A.L. (2013). Impact of peripheral levels of chemokines, BDNF and oxidative markers on cognition in individuals with schizophrenia. J. Psychiatr. Res..

[283] Ishizuka K., Fujita Y., Kawabata T., Kimura H., Iwayama Y., Inada T., Okahisa Y., Egawa J., Usami M., Kushima I. (2017). Rare genetic variants in CX3CR1 and their contribution to the increased risk of schizophrenia and autism spectrum disorders. Transl Psychiatry.

[284] Gao X., Mi Y., Guo N., Xu H., Jiang P., Zhang R., Xu L., Gou X. (2018). Glioma in Schizophrenia: Is the Risk Higher or Lower?. Front. Cell. Neurosci.

[285] García-Cuesta E.M., Santiago C.A., Vallejo-Díaz J., Juarranz Y., Rodríguez-Frade J.M., Mellado M. (2019). The Role of the CXCL12/CXCR4/ACKR3 Axis in Autoimmune Diseases. Front. Endocrinol. (Lausanne).

[286] Malmqvist A., Schwieler L., Orhan F., Fatouros-Bergman H., Bauer M., Flyckt L., Cervenka S., Engberg G., Piehl F., Erhardt S. (2019). Increased peripheral levels of TARC/CCL17 in first episode psychosis patients. Schizophr. Res..

[287] Laurikainen H., Vuorela A., Toivonen A., Reinert-Hartwall L., Trontti K., Lindgren M., Keinänen J., Mäntylä T., Paju J., Ilonen T. (2020). Elevated serum chemokine CCL22 levels in first-episode psychosis: Associations with symptoms, peripheral immune state and in vivo brain glial cell function. Transl. Psychiatry.

[288] Hill S.L., Shao L., Beasley C.L. (2020). Diminished levels of the chemokine fractalkine in post-mortem prefrontal cortex in schizophrenia but not bipolar disorder. World J. Biol. Psychiatry.

[289] Chamera K., Szuster-Głuszczak M., Trojan E., Basta-Kaim A. (2020). Maternal Immune Activation Sensitizes Male Offspring Rats to Lipopolysaccharide-Induced Microglial Deficits Involving the Dysfunction of CD200-CD200R and CX3CL1-CX3CR1 Systems. Cells.

[290] Zhou H., Wang J., Zhang Y., Shao F., Wang W. (2020). The Role of Microglial CX3CR1 in Schizophrenia-Related Behaviors Induced by Social Isolation. Front. Integr. Neurosci..

[291] Cathomas F., Klaus F., Guetter K., Chung H.K., Raja Beharelle A., Spiller T.R., Schlegel R., Seifritz E., Hartmann-Riemer M.N., Tobler P.N. (2021). Increased random exploration in schizophrenia is associated with inflammation. NPJ Schizophr.

[292] Ranasinghe R., Eri R. (2018). Pleiotropic Immune Functions of Chemokine Receptor 6 in Health and Disease. Medicines.

[293] Na K.S., Jung H.Y., Kim Y.K. (2014). The role of pro-inflammatory cytokines in the neuroinflammation and neurogenesis of schizophrenia. Prog. Neuropsychopharmacol. Biol. Psychiatry.

[294] Fillman S.G., Sinclair D., Fung S.J., Webster M.J., Shannon Weickert C. (2014). Markers of inflammation and stress distinguish subsets of individuals with schizophrenia and bipolar disorder. Transl. Psychiatry.

[295] Boerrigter D., Weickert T.W., Lenroot R., O’Donnell M., Galletly C., Liu D., Burgess M., Cadiz R., Jacomb I., Catts V.S. (2017). Using blood cytokine measures to define high inflammatory biotype of schizophrenia and schizoaffective disorder. J. Neuroinflamm..

